# Beyond the MUN domain, Munc13 controls priming and depriming of synaptic vesicles

**DOI:** 10.1016/j.celrep.2024.114026

**Published:** 2024-05-21

**Authors:** Jeremy Leitz, Chuchu Wang, Luis Esquivies, Richard A. Pfuetzner, John Jacob Peters, Sergio Couoh-Cardel, Austin L. Wang, Axel T. Brunger

**Affiliations:** 1 Department of Molecular and Cellular Physiology, Stanford University, Stanford, CA, USA; 2 Department of Neurology and Neurological Sciences, Stanford University, Stanford, CA, USA; 3 Department of Structural Biology, Stanford University, Stanford, CA, USA; 4 Department of Photon Science, Stanford University, Stanford, CA, USA; 5 Howard Hughes Medical Institute, Stanford University, Stanford, CA, USA; 6 Lead contact

## Abstract

Synaptic vesicle docking and priming are dynamic processes. At the molecular level, SNAREs (soluble NSF attachment protein receptors), synaptotagmins, and other factors are critical for Ca^2+^-triggered vesicle exocytosis, while disassembly factors, including NSF (*N*-ethylmaleimide-sensitive factor) and α-SNAP (soluble NSF attachment protein), disassemble and recycle SNAREs and antagonize fusion under some conditions. Here, we introduce a hybrid fusion assay that uses synaptic vesicles isolated from mouse brains and synthetic plasma membrane mimics. We included Munc18, Munc13, complexin, NSF, α-SNAP, and an ATP-regeneration system and maintained them continuously—as in the neuron—to investigate how these opposing processes yield fusogenic synaptic vesicles. In this setting, synaptic vesicle association is reversible, and the ATP-regeneration system produces the most synchronous Ca^2+^-triggered fusion, suggesting that disassembly factors perform quality control at the early stages of synaptic vesicle association to establish a highly fusogenic state. We uncovered a functional role for Munc13 ancillary to the MUN domain that alleviates an α-SNAP-dependent inhibition of Ca^2+^-triggered fusion.

## In brief

Leitz et al. present an assay that uses endogenous synaptic vesicles and reproduces many aspects of synaptic vesicle fusion. When presynaptic proteins are maintained throughout the assay, several factors play a quality control role in establishing a highly fusogenic state, and domains ancillary to the MUN domain of Munc13 are critical for this role.

## Graphical Abstract

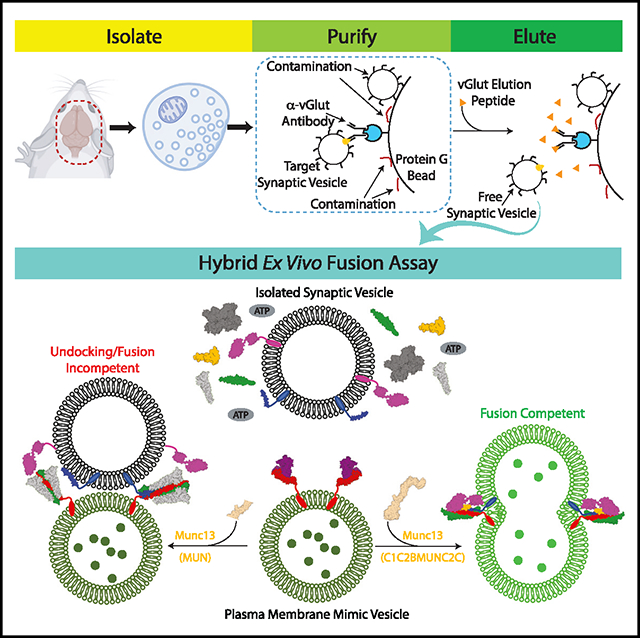

## INTRODUCTION

Synaptic vesicle fusion with the plasma membrane is the molecular process underlying chemical neurotransmission. The neuronal SNARE (soluble NSF attachment protein receptor) proteins syntaxin-1, SNAP-25 (synaptosomal-associated protein 25), and synaptobrevin-2/VAMP2 (vesicle-associated membrane protein 2)^1^ form the *trans* ternary SNARE complex^2^ that brings the plasma membrane and the membrane of the synaptic vesicle into close apposition and ultimately provides the energy to fuse these two membranes and release synaptic vesicle contents into the synaptic cleft.^3^ The SM (Sec1/Munc18-like) proteins Munc18 and Munc13 organize SNARE complex assembly,^4,5^ ensuring a functional parallel SNARE complex.^6^ Once the *trans* SNARE complex is assembled correctly, the Ca^2+^ sensor synaptotagmin-1 (Syt1) binds to the *trans* SNARE complex^7,8^ and couples Ca^2+^ influx to rapid neurotransmitter release.^9,10^ The soluble accessory protein complexin also binds to the SNARE complex^11^ and inhibits premature full zippering of the ternary SNARE complex and consequent membrane fusion, thus holding the vesicle in a primed-for-fusion state.^7,8,12,13^

Following fusion, the AAA+ protein NSF (*N*-ethylmaleimide sensitive factor),and the adaptor protein α-SNAP (soluble NSF attachment protein) unwind and separate the SNARE complex into its components,^14^ allowing synaptic vesicle arrival, *trans* SNARE complex assembly, and ultimately fusion to repeat. In addition, NSF and α-SNAP disassemble the *cis* binary (syntaxin-1/SNAP-25) complex,^4,6,15^ cis syntaxin tetramers (K.I. White, Y.A. Khan, J. Diao, K. Qiu, S.C.-C., R.A.P., L.E., and A.T.B., unpublished data), and *trans* SNARE complexes under some conditions.^16^ Munc18 captures the liberated syntaxin molecules,^17^ setting the stage for SNARE assembly via Munc13.^4,6,15^ Because all these components are always present in the presynaptic terminal, the question arises as to how the machinery directs the system toward triggered synaptic vesicle fusion and how it regulates presynaptic short-term plasticity by priming, transient association, and depriming.^18–21^

Direct investigation of the individual protein components involved in live neurons is challenging owing to the inherent complexity of the cellular milieu. Here, we expand on a previous *in vitro* assay that permits the systematic investigation of the roles of individual proteins (e.g., complexin and Munc13).^6,22,23^ We isolated and purified intact glutamatergic synaptic vesicles (ISVs) from mouse brains and developed an *ex vivo/in vitro* assay to measure Ca^2+^-triggered fusion between the ISVs and plasma membrane-mimicking vesicles containing reconstituted syntaxin-1 and SNAP-25. Our system not only allows the addition or subtraction of individual components during various stages of vesicle association and fusion, but it can also distinguish between synaptic vesicle association and Ca^2+^-triggered synaptic vesicle fusion. We included complexin, NSF, α-SNAP, Munc18, Munc13, and an ATP-regeneration system, making this assay a relatively complete reconstituted assay for studying synaptic vesicle fusion and other aspects of the synaptic vesicle cycle. We find that the continuous presence of NSF, α-SNAP, and ATP antagonized fusion in the presence of the catalytic MUN domain of Munc13, but the larger C1C2BMUNC2C fragment was able to alleviate this inhibitory effect and achieve the highest Ca^2+^-triggered fusion synchrony. Thus, our hybrid fusion assay provided mechanistic insight into physiologically relevant questions, such as why the MUN domain incompletely rescues Munc13 deletion.^24,25^

## RESULTS

### Distinguishing different modes of ISV behavior

**Figure 1.**
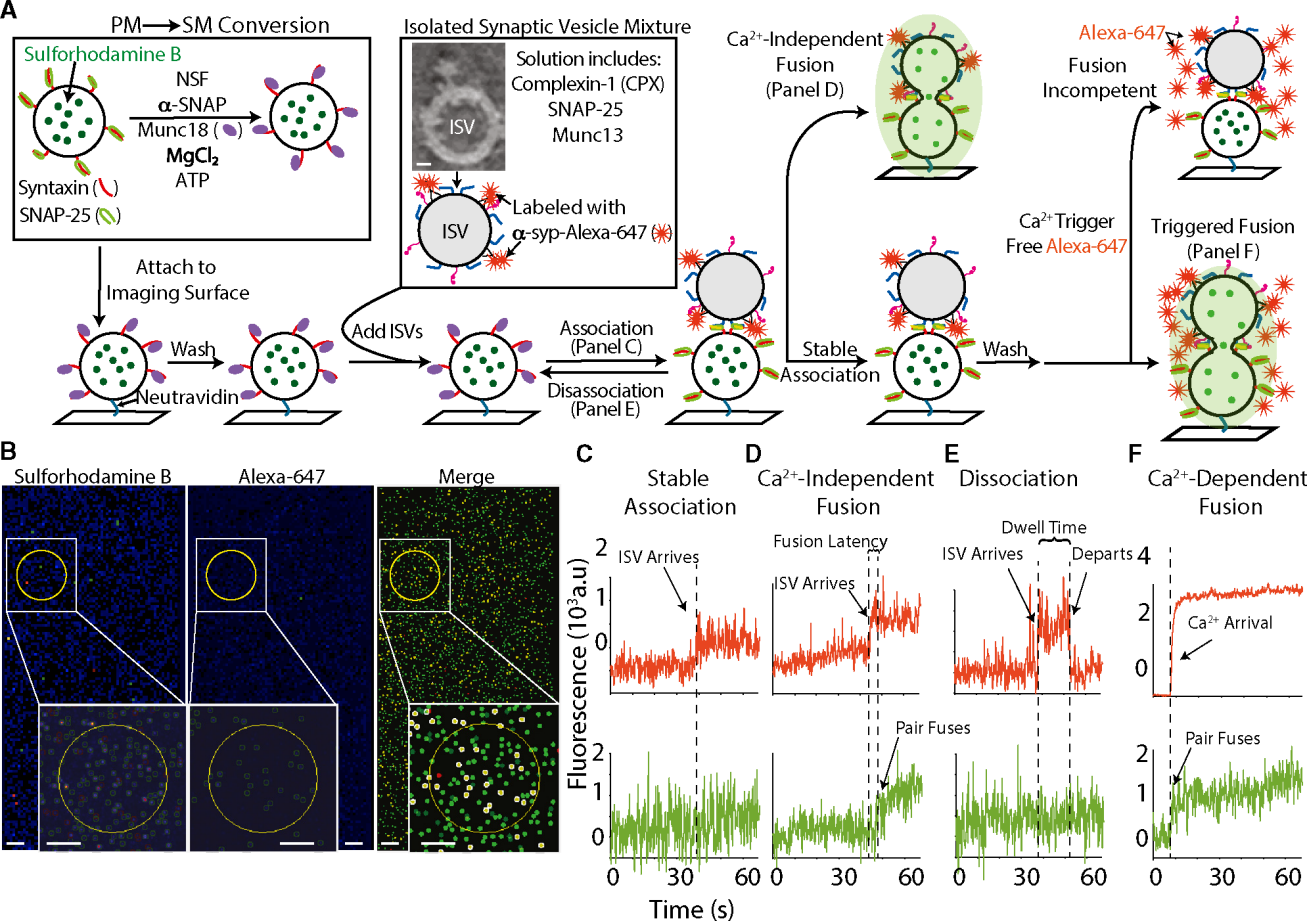
Experimental design of the ISV-SM system and representative imaging data (A) Overview of the experimental scheme (see Tables S1 and S2 and STAR Methods for more details). (B) Example images. (Left) Green channel for monitoring the sulforhodamine B, (middle) red channel for monitoring Alexa 647, and (right) merge where regions of interest (ROIs) have been falsely colored (green for the sulforhodamine B fluorescence intensity, red for Alexa 647 fluorescence intensity, and yellow for overlapping signals). Scale bars, 10 μm. (C–F) Example traces of stable association (C), Ca^2+^-independent fusion (D), dissociation (E), and Ca^2+^-dependent fusion (F).

We isolated and purified fusion-competent ISVs from mouse brains (Figures S1–S3 and STAR Methods; see also Leitz et al.^26^). To mimic the plasma membrane, we used proteoliposomes termed SM (for Sec1/Munc18- or Syntaxin/Munc18-containing) vesicles. Our SM reconstitutions began with proteoliposomes containing only the reconsituted proteins syntaxin and SNAP-25 (termed PM vesicles), and encapsulating the self-quenching dye sulforhodamine B. The binary syntaxin-1-SNAP-25 complex is then disassembled by addition of NSF and α-SNAP, in the presence of Munc18, Mg^2+^, and ATP^4,6^ (Figure 1A). We found this PM-to-SM vesicle conversion necessary, as the direct formation of the full-length syntaxin-1-Munc18 complex is difficult in the presence of certain detergents.^6,27^ We first attached SM vesicles to the imaging surface via a biotin-neutravidin-biotin interaction and washed unattached SM vesicles away. Separately, ISVs were labeled with a fluorescent antibody (anti-synaptophysin-Alexa-647, abbreviated as α-syp-Alexa-647; STAR Methods) and added along with accessory proteins to the imaging chamber before beginning image acquisition (Figure 1A). ISV-SM vesicle association was detected as a stepwise increase in the “red” channel that monitors the Alexa-647 fluorescence intensity, while any fusion that may occur during association was detected in the “green” channel that monitors the sulforhodamine B fluorescence intensity (Figures 1B–1D). We also observed association events in which a vesicle would arrive, remain for a period, and then depart. We labeled these as “dissociation” events and measured the duration “dwell time” when vesicles were associated (Figure 1E) with a minimum dwell time cutoff of 4 s. After association, the channel was washed using a buffer containing the specified components. Finally, the same buffer with addition of 50 μM Ca^2+^ and free Alexa-647 dye was injected to trigger fusion (Figures 1A and 1F). Fusion events were detected by monitoring the fluorescence intensity of sulforhodamine B, while Ca^2+^ arrival was detected by the fluorescence intensity of the free Alexa-647 dye (Figure 1F). In the first set of experiments, we used the catalytic MUN fragment of Munc13 and later tested the effect of using a longer fragment of Munc13. The schema shown in Figure 1A generally applies to all experiments described in this work. More detailed schemas are provided for each experiment, with changes marked in bold and/or red.

### Effect of PM-to-SM vesicle conversion

**Figure 2.**
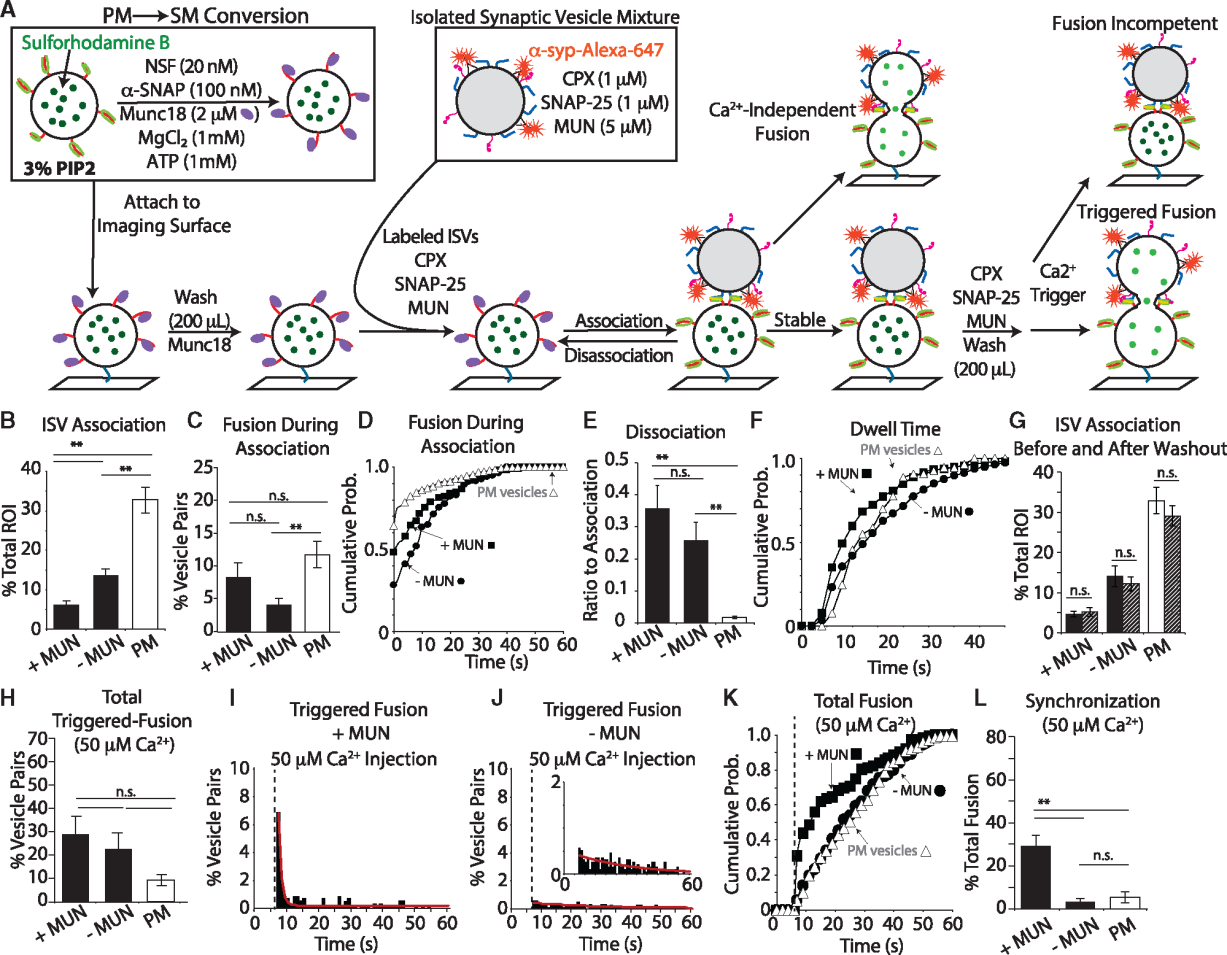
Fusion assay with ISV and SM (or PM) vesicles (A) Experimental scheme for the ISV-SM system (see Tables S1 and S2 and STAR Methods for more details). For the ISV-PM system, see Figures S2 and S3. (B) ISV association in the ISV-SM system (black bars) compared to the simple ISV-PM system (white bar, see Figure S2). (C) Quantification of total fusion during association. (D) Cumulative probability histogram of the fusion latency shows a longer latency in the ISV-SM system exacerbated by the absence of MUN (Kolmogorov-Smirnov [KS] test p value = 0.017 for all SM-PM comparisons, KS test p value = 0.6 comparing with and without MUN). (E) Quantification of dissociation in the ISV-SM system (black bars) and PM system (white bar). MUN did not affect dissociation. (F) Cumulative probability distribution of dwell times after a minimum association time of 4 s (KS test p value <0.01 for all comparisons). (G) ISV association at the end of the association period (solid bars) and after a 5-min incubation followed by washout (striped bars). Note that no incubation was performed in the case of the ISV-PM system because of the high vesicle association rate. Data from panel B are included for direct comparison. (H) Quantification of total vesicle fusion triggered by 50 μM Ca^2+^ injection. See also Figure S3E. (I and J) Distribution of fusion times after Ca^2+^ injection (dashed line) in the presence of MUN (I) and absence of MUN (J). Red lines are the sum of two exponential decays fit by the sum of least-squares minimization. See also Figures S3C and S3D. (K) Cumulative probability distribution of data in (I) and (J) (black squares and circles, respectively) and, for comparison, the ISV-PM vesicle system in response to50 μM Ca^2+^ injection (white triangles). The +MUN condition was significantly faster than both the –MUN and the ISV-PM conditions (KS test p value <0.001 for both comparisons). There was no significant difference in the distribution between the –MUN condition and the PM vesicles (KS test p value = 0.93). See also Figure S3. (L) The ratio of fusion events in the first second (5 acquisition frames) after Ca^2+^ arrival to all fusion events (synchronization). ISV association, dissociation, and dissociation dwell times are normalized to the total number of available SM/PM vesicles. All fusion data, both Ca^2+^-dependent and -independent, are normalized to the total number of vesicle pairs. Data are presented as mean ± SEM for association, fusion during association, and dissociation experiments consisting of multiple videos as replicates (Tables S1 and S2). For all bar graphs, a one-way ANOVA with post hoc Tukey-Kramer analysis was performed. *p < 0.05, and **p < 0.005; n.s., not significant.

After tethering SM vesicles to the imaging surface (Figure 2A), we washed them with “vesicle buffer” (20 mM HEPES pH 7.4, 90 mM NaCl, 20 μM EGTA) containing Munc18 to remove unbound SM vesicles and PM-to-SM conversion protein components (NSF, α-SNAP, and ATP). Next, we injected labeled ISVs, 1 μM complexin, 1 μM SNAP-25, 500 μM tris(2-carboxyethyl) phosphine (TCEP), and either 5 μM MUN or no MUN, and monitored the association between ISVs and SM vesicles (Figure 2B). Curiously, in the absence of MUN, more vesicle pairs associated in the ISV-SM system. Moreover, in the simpler ISV-PM system (consisting of only ISVs and PM vesicles, i.e., without any of the other components), even more vesicle associations occurred (Figures S2H and 2B). Thus, ISVs are substantially slower to associate with SM vesicles than simpler PM vesicles.

During association, a subset of vesicles fuses in the absence of Ca^2+^. We quantified the number of vesicle pairs that fused and the time between association and fusion for each pair, which we term “latency” (Figures 2C and 2D, respectively). For the ISVSM system, in the presence of MUN, 8.5% ± 0.8% of the total number of vesicle pairs fused, while in the absence of MUN, fusion was not significantly different (p = 0.3). Analysis of the latency between ISV arrival and fusion showed that in the presence of MUN, ~48% of the fusion events occurred within the first second of vesicle association. Without MUN, this proportion dropped to ~29% (Figures 2D and S4A). We note that fusion latency was independent of vesicle arrival time (Figure S5).

ISV-SM association is reversible, and we observe transient association events in which a vesicle arrives at an SM vesicle, remains for a period, and then disappears (Figure 1E). To exclude events that were due to ISVs passing by the imaging surface, we defined an arbitrary cutoff time of ~4 s (STAR Methods). We normalized dissociation to the number of vesicles that were stably associated (Figure 2E). Thus a value of 1 would indicate that for every pair that stably associated, another pair dissociated. A value of zero would indicate there were no observed dissociation events within the imaging period. In the presence of MUN, the ratio of dissociation-to-association events was 0.36 ± 0.07 (Figure 2E); that is, more than one-third as many ISVs transiently associated as stably associated. In the absence of MUN, this ratio was slightly, but not significantly, reduced to 0.26 ± 0.06. In the simpler ISV-PM system, we rarely observed dissociation events, with a ratio of 0.02 ± 0.003 (Figures 2E [white bar] and S2L). Thus, in the ISV-SM system, we observed a substantial proportion of vesicle pairs that underwent dissociation regardless of the presence or absence of MUN. We also quantified the dwell time of the association of an ISV at an SM/PM vesicle (Figure 2F). Dwell-time distributions could be fit with a gamma distribution (Figures S4B and S4B). We present them as cumulative probability histograms to allow easier direct comparison of distributions (Figure 2F). In the absence of MUN, the dwell-time distribution skewed longer, meaning that ISVs resided at SM vesicles for more extended periods before departing compared to when MUN was included (Figure 2F).

Due to the low rate of ISV association and fusion in the ISV-SM system, we allowed ISVs to form pairs for an additional 5 min. After washout, we again quantified the ISV-SM vesicle pair number (Figure 2G). The longer association period did not alter the total number of associated vesicles in the presence or absence of MUN. Finally, the simpler ISV-PM system also had a similar number of pairs associated (Figures 2G and S2H). Of note, we did not use a 5-min incubation period for the simpler ISV-PM system because the rate of association is relatively high, and a wash was performed immediately after association. These data suggest that the process governing ISV-SM vesicle association is independent of the presence of MUN, although MUN alters the distribution of dissociation dwell times.

Next, we triggered fusion by injecting 50 μM Ca^2+^ in vesicle buffer supplemented with 1 μM complexin, 1 μM SNAP-25, 500 μM TCEP, 100 ng Alexa-647 dye (as an indicator of Ca^2+^ arrival) (Figures 1A and 1F), and with 5 μM MUN or no MUN. We measured several aspects of Ca^2+^-triggered vesicle fusion: the total number of vesicles that fused (Figure 2H), the distribution of fusion (Figures 2I–2K), and the number of vesicles that fused within the first second (5 acquisition frames) of Ca^2+^ arrival as indicated by the arrival of free Alexa-647 (Figure 2L). This first measure assesses gross fusion competence, while the latter measures fusion synchronicity. Surprisingly, we did not observe any significant difference in gross fusion competence for the ISV-SM system with and without MUN (Figure 2H). In contrast, the simpler ISV-PM system contained few fusogenic vesicle pairs (Figures 2H, S3C, and S3E).

We did observe a significant difference in the distribution of fusion events and synchronization of fusion. In the ISV-SM system, when MUN was included, a substantial portion of fusion events occurred immediately upon Ca^2+^ arrival. The distribution of fusion events was fit by the sum of two exponential decay functions: a dominant fast component with a time constant of 1.5 s^−1^ and a slow component with a time constant of 0.04 s^−1^ (Figure 2I, red line). By comparison, fusion in the absence of MUN was highly desynchronized and fusion occurred as a single exponential decay with a time constant of 0.036 s^−1^ (Figure 2J, red line), similar to the ISV-PM system (Figures 2K, S3F, and S3G). In the presence of MUN, the first second (5 acquisition frames) after Ca^2+^ arrival included 29.1% ± 5.2% of all fusion events, compared to only 3.2% ± 1.6% fusion events in the absence of MUN and 5.4% ± 2.5% in the simple ISV-PM system (Figure 2L).

Together, these experiments indicate that compared to the simpler ISV-PM system, ISVs are slower to associate with SM vesicles, and these associations are more tenuous and prone to reversal (dissociation). However, the ISVs that do stably associate are more homogeneous in their response to Ca^2+^ influx (more synchronous). While the increase in synchronization was expected, since the ISV-SM system is more complete, we were surprised to observe such a disparity in the vesicle association rate and the substantial increase in dissociation events. We were also surprised to see no difference in Ca^2+^-independent fusion during association, even when MUN was excluded, and no difference in total fusion. One explanation for the apparent decrease in vesicle association for the ISV-SM system may lie in a convolution of protein-protein and protein-lipid interactions. Another non-mutually exclusive possibility is that the PM-to-SM conversion is incomplete, resulting in a mixture of SNARE complexes,^6^ and only a subset is fusion competent. Therefore, we sought to test these two possibilities in the following experiments.

### PI(4,5)P2 concentration modulates fusion efficiency

**Figure 3.**
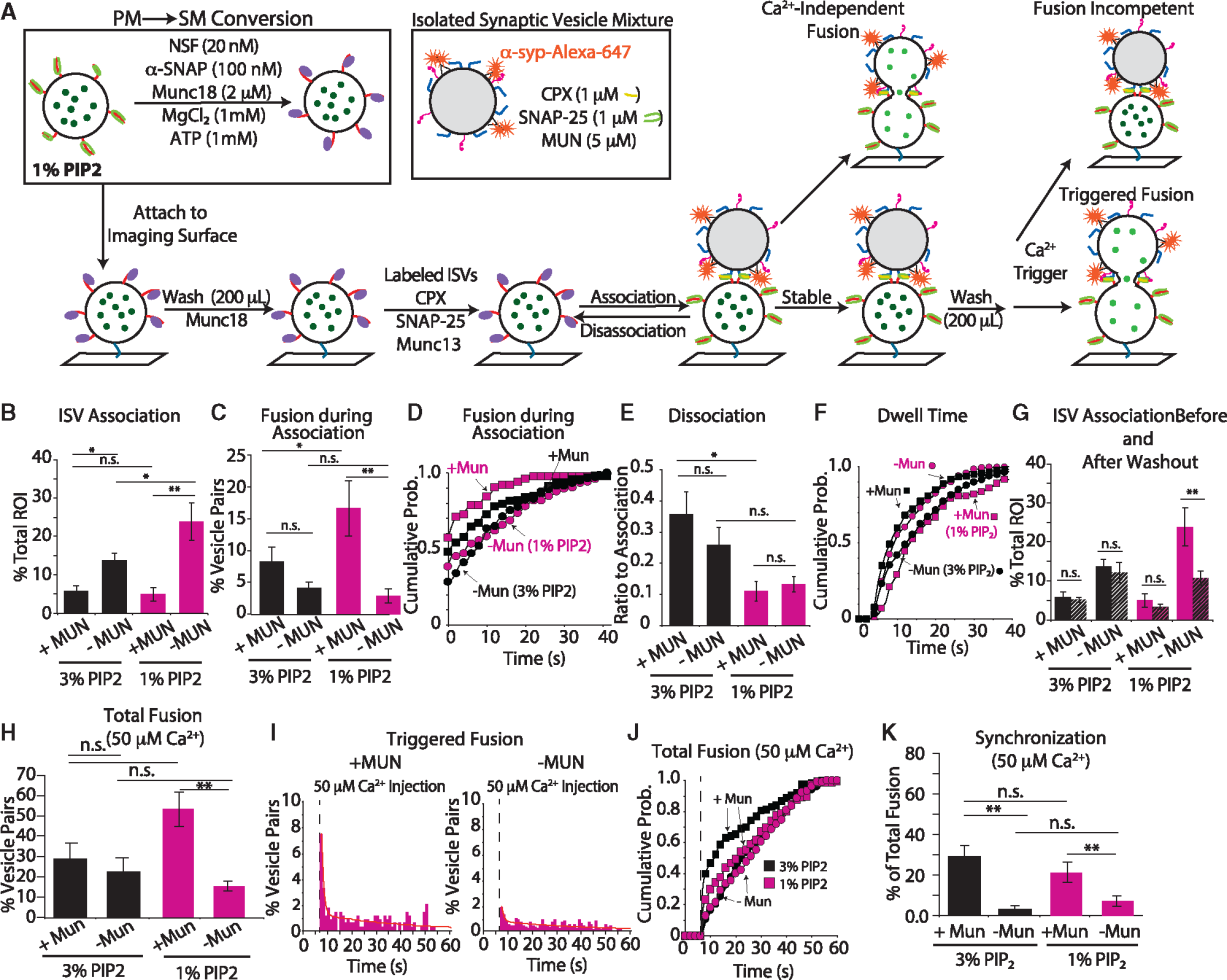
Effect of PIP2 concentration in SM vesicles (A) Experimental scheme. The specified PIP2 concentration is used for the PM vesicles prior to SM conversion (see Tables S1 and S2 and STAR Methods for more details). (B–D) Quantification of ISV-SM association (B), fusion during association (C), and the distribution of latency time between association and fusion (D) with and without MUN in both 1% (magenta) and 3% (black) PIP2 conditions (see Tables S1 and S2 and STAR Methods for more details). (E and F) Quantification of dissociation events (E) and cumulative probability distribution of dwell times after a minimum association time of 4 s (F) with and without MUN for 1% PIP2 (magenta) and, for reference, 3% PIP2 (black) conditions. In the 1% PIP2 system, MUN increased the dwell times, shifting the distribution to the right (KS test p value <0.05 for all comparisons). The 3% PIP2 conditions are shown in black for comparison. (G) Quantification of the association after the association period (solid bars) and after a 5-min incubation followed by washout (striped bars) showed a difference only in the 1% PIP2 system when MUN was excluded. Data from panel B are included for direct comparison. (H–K) Quantification of Ca^2+^-triggered fusion. (H) Total fusion in response to 50 μM Ca^2+^ injection at 3% PIP2 (black bars) and 1% PIP2 (magenta bars) in the SM system. (I) Fusion histograms of 1% PIP2 fusion with and without MUN in response to Ca^2+^ arrival (dashed line); the red line is the sum of two exponential decay functions fit by least-squares minimization. (J) Cumulative probability histogram of Ca^2+^-triggered fusion for experiments at 1% PIP2 (magenta squares and circles) and 3% PIP2 (black squares and circles). The 1% PIP2 with MUN distribution (magenta squares) is not significantly different from that without MUN (magenta circles) or 3% PIP2 without MUN distribution (KS test p value >0.1 for all comparisons). However, all conditions were significantly reduced from the 3% PIP2 system with MUN (KS test p value <0.05 for all comparisons). (K) The synchronization or ratio of fusion events in the first second (5 acquisition frames) after Ca^2+^ arrival to all fusion events. ISV-SM association and association after washout are normalized to the total number of available SM vesicles (ROIs). Both Ca^2+^-dependent and -independent fusion data are normalized to the total number of vesicle pairs. Dissociation is normalized to the association. Synchronization is normalized to the total number of fusion events. Data are presented as mean ± SEM for association, fusion during association, and dissociation measurements for several replicates (Tables S1 and S2). For all bar graphs, a one-way ANOVA with post hoc Tukey-Kramer analysis was performed; *p < 0.05, **p < 0.005; n.s., not significant. For all cumulative probability histograms, a KS test was performed for all pairwise comparisons.

Synaptotagmin-1 interacts not only with the SNARE complex^7,8^ but also with PIP2 via electrostatic interactions with the polybasic region of the C2B domain of synaptotagmin-1,^28–34^ and this latter interaction might act to facilitate fusion upon Ca^2+^ triggering.^35^ While the exact concentration of PIP2 at the active zone is not known, measurements by thin-layer chromatography coupled with high-performance liquid chromatography suggest that PIP2 constitutes ~1% of total phospholipid in cultured cortical neurons, albeit PIP2 is asymmetrically distributed in the plasma membrane.^36,37^ To test the influence of PIP2 in our system, we decreased the PIP2 concentration of the SM vesicles from 3% to 1% and repeated our docking and fusion experiments (Figure 3A).

ISV association with 1% PIP2 was like the 3% PIP2 condition where again, in the absence of MUN, we observed more ISV vesicle association (Figure 3B, magenta). Ca^2+^-independent fusion during association had a more pronounced difference in the 1% PIP2 condition (Figures 3C and S4C). Pairs that fused did so slightly sooner in the 1% PIP2 system with and without MUN compared to the 3% PIP2 system (Figure 3D). Vesicle dissociation in the 1% PIP2 system was independent of MUN and substantially reduced compared to the 3% PIP system (Figure 3E). The dwell times of dissociation were reversed compared to the 3% PIP2 system, where excluding MUN now resulted in shorter ISV dwell times (Figures 3F and S4D). Before Ca^2+^ triggering, we reexamined the number of associated ISV-SM pairs after washout. In the 1% PIP2 system with no MUN, there was a significant difference in the number of pairs remaining after washing (Figure 3G). This suggests that this condition produced interactions that were susceptible to removal by washing.

Next, we evaluated triggered fusion. Although we did not observe a significant increase in the total number of vesicles that fused between the 3% PIP2 system and the 1% PIP2 system (Figure 3H), fusion with and without the MUN domain was now significantly different in the reduced 1% PIP2 system. As expected, the fusion kinetics after Ca^2+^ entry was slower at 1% PIP2 than at 3% PIP2 (Figures 3I and 3J), consistent with previous results.^34^ In the presence of MUN, 21.1% ± 5.0% of total pairs fused within the first second of Ca^2+^ arrival, and the distribution of fusion events could be described by the sum of two exponential decays with decay constants 1.2 s^−1^ (contributing 83%) and 0.03 s^−1^ (Figure 3I, left). The kinetics were slower in the absence of MUN, with 7.3% ± 2.7% of pairs fusing within the first second, and the distribution was fit with decay constants 1.1 s^−1^ (61%) and 0.03 s^−1^ (Figure 3I, right). Therefore, while PIP2 does not affect the total ISV-SM vesicle association, the associations that do form in 1% PIP2 are more stable (i.e., there is less dissociation). Moreover, the PIP2 concentration alters total fusion (Figure 3H) and the overall distribution of fusion time in the presence of MUN (Figures 3I and 3J), but there is no significant change in synchronization.

### An ATP-regeneration system increases fusion synchronicity

**Figure 4.**
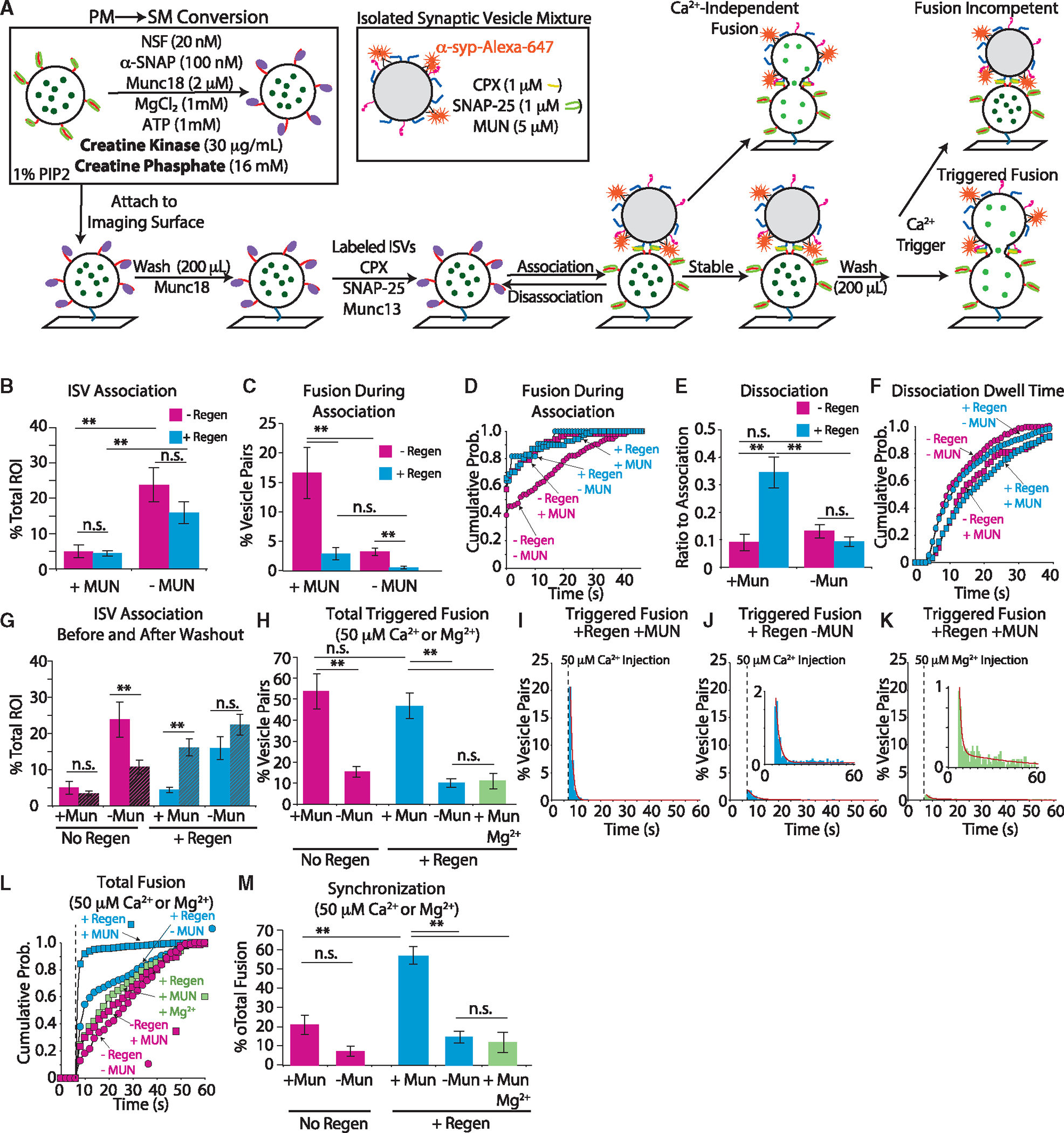
Adding an ATP-regeneration system increases Ca^2+^-triggered fusion and synchrony (A) Experimental scheme (changes are indicated in bold). PM vesicles are converted to SM vesicles in bulk with the ATP-regeneration system present before addition to the imaging slide (see Tables S1 and S2 and STAR Methods for more details). (B–D) Quantification of ISV-SM vesicle association (B), fusion during association (C), and fusion latency (D) in the presence (blue) and absence (magenta) of the regeneration system (abbreviated “+/− Regen”). Addition of the regeneration system skewed fusion latencies smaller and made them similar to the MUN condition without the regeneration system (KS test p value >0.1 for both with and without MUN). Only when the regeneration system was not included and without MUN was the latency distribution significantly different (KS test p value <0.05 for all comparisons). (E and F) Quantification of ISV dissociation events (E) and ISV cumulative probability histogram of dwell times after a minimum association time of 4 s (F). The regeneration system in the presence of MUN greatly increased the prevalence of dissociation events. The dwell time before dissociation was unaffected by the regeneration system (KS test p value >0.1 for relevant comparisons), but was still MUN dependent (KS test p value <0.05). (G) Quantification of vesicles that remained associated after the association period (solid bars) and after a 5-min incubation followed by washout (striped bars) with and without the regeneration system (blue and magenta, respectively). Data from panel B are included for direct comparison. (H) Quantification of Ca^2+^-triggered fusion in all systems tested. (I) Histogram of triggered fusion after Ca^2+^ injection (dashed line); red line is a single exponential fit. (J) Histogram of Ca^2+^-triggered fusion in the absence of MUN; red line is a sum of two exponential decays. (K) Histogram of triggered fusion latency after Mg^2+^ injection in the presence of MUN (dashed line); red line is a sum of two exponential decay functions (with decay rate constants of 0.7 s^−1^ and 0.03 s^−1^). (L) Cumulative probability distributions of Ca^2+^-triggered fusion. The regeneration system shifted fusion distributions closer to Ca^2+^ arrival (KS test p value <0.05 for both with and without MUN compared to triggering with Mg^2+^ and without the regeneration system). Including MUN shifted the distribution further (KS test p value <0.05 with MUN compared to no MUN). (M) Ratio of fusion events in the first second (5 acquisition frames) after Ca^2+^ or Mg^2+^ arrival to all fusion events (referred to as synchronization). ISV-SM vesicle association is normalized to the total number of available SM vesicles (“total ROI”). All fusion data, both Ca^2+^-dependent and Ca^2+^-independent, are normalized to the total number of ISV-SM vesicle pairs. Data are presented as mean ± SEM for association, fusion during association, and dissociation measurements for several replicates (Tables S1 and S2). For all bar graphs, a one-way ANOVA with post hoc Tukey-Kramer analysis was performed; *p < 0.05, **p < 0.005; n.s., not significant. For all cumulative probability histograms, a KS test was performed for all pairwise comparisons.

All the above experiments started with a defined ATP concentration that is expected to deplete rapidly as NSF undergoes hydrolysis cycles. To maintain a constant ATP concentration within a physiological level, we introduced an established ATP-regeneration system wherein creatine kinase and creatine phosphate replenish ATP,^38,39^ resulting in an approximately constant ATP concentration (Figure 4A). In this modified scheme, NSF and α-SNAP activity, and thus PM-to-SM conversion, should be prolonged.

In the presence of 5 μM MUN, there was no substantial difference in ISV-SM vesicle association with the ATP-regeneration system (Figure 4B). In the absence of MUN, there was still more association with the ATP-regeneration system, and it was not substantially altered by the regeneration system itself. Intriguingly, the ATP-regeneration system did have a profound impact on Ca^2+^-independent fusion during association, largely suppressing it when included (Figures 4C and S4E). Moreover, in the absence of MUN with the regeneration system, almost no Ca^2+^-independent fusion occurred. Of those pairs that did fuse during ISV-SM association with the regeneration system, a similar fraction fused immediately upon arrival in both the presence and absence of MUN, respectively (Figures 4D and S4E).

ISV-SM dissociation substantially increased, by more than 4-fold, in the presence of the ATP-regeneration system when MUN was included (Figure 4E). Despite the substantial increase in dissociation occurrences, the distribution of dwell times was unchanged by the regeneration system (Figure 4F). Examination of ISV-SM vesicle association after 5-min incubation and washout revealed that not only did ISVs remain associated but now further association had occurred (Figure 4G).

For Ca^2+^-triggered fusion, the total amount of triggered fusion was comparable to fusion in the absence of the ATP-regeneration system (Figure 4H, blue). However, the fusion kinetics was dramatically faster with the ATP-regeneration system. Ca^2+^-triggered fusion now proceeded via single exponential decay with decay constant 1.3 s^−1^. Nearly all fusion was complete after 2 s (Figures 4I, 4L, and 4M). As expected, this Ca^2+^-triggered fusion activity was MUN dependent. The triggered fusion without MUN that did occur was still somewhat synchronous with Ca^2+^ arrival with a decay described by the sum of two exponential functions, with a fast component of 0.4 s^−1^ (contributing 95%) and a slow component of 0.01 s^−1^ (Figures 4J, 4L, and 4M). As a control, we injected 50 μM Mg^2+^ in the presence of MUN and found that only a minimal amount of fusion could be elicited (Figures 4K–4M).

Taken together, inclusion of the ATP-regeneration system results in ISV-SM pairs that are more synchronous in their sensitivity to Ca^2+^ influx. However, even in this modified scheme (Figure 4A), some components (in particular, NSF and α-SNAP) were removed by washing before triggering fusion, whereas in the neuron these components are continuously present. Therefore, to bring our fusion assay closer to physiological conditions, we next kept all components present.

### Continuous presence of α-SNAP and MUN

**Figure 5.**
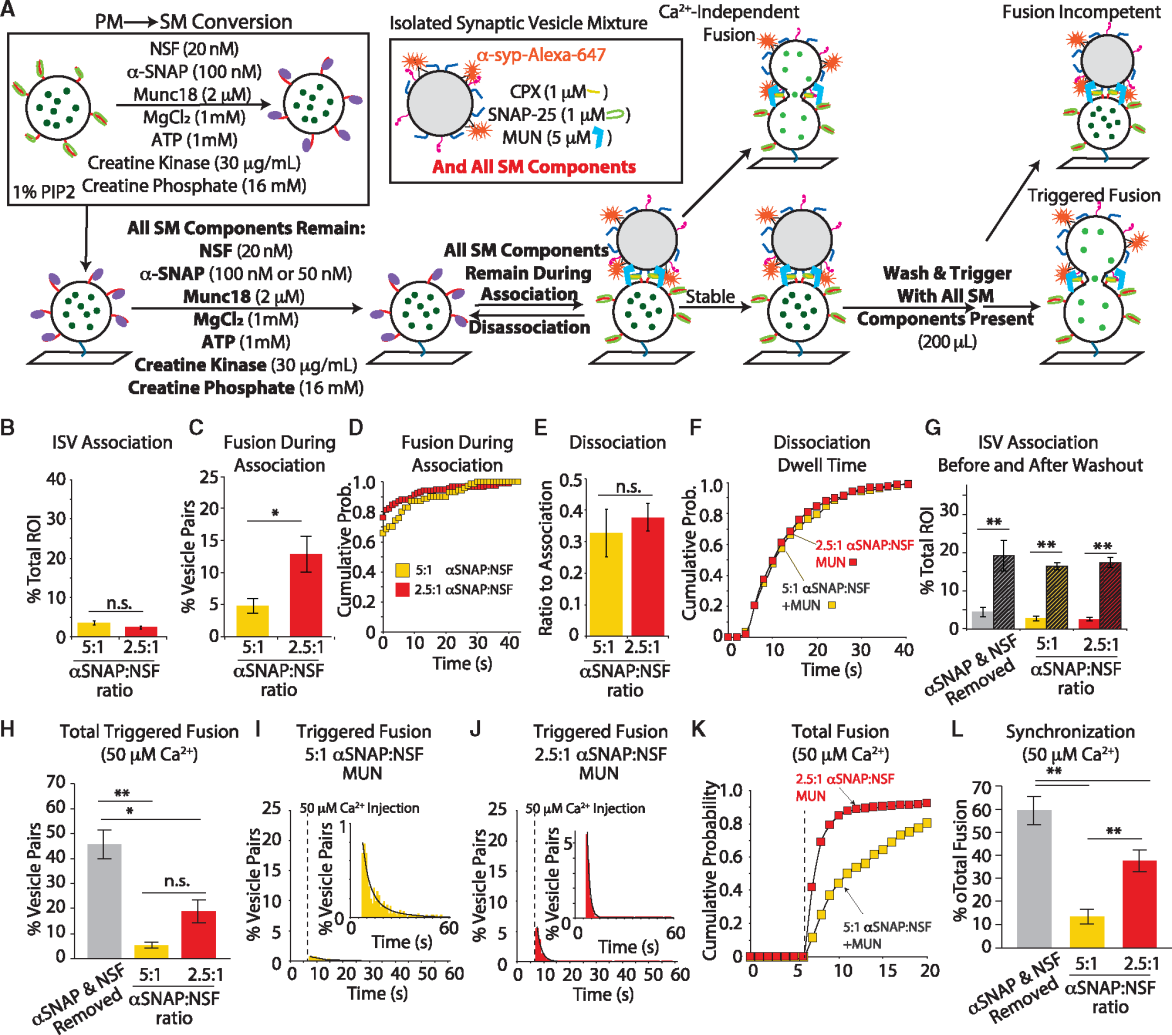
α-SNAP inhibits and desynchronizes fusion but does not alter ISV association of dissociation (A) Experimental scheme (changes to scheme in Figure 4A are indicated in bold and bold red). PM vesicles are converted to SM vesicles with the ATP-regeneration system and the regeneration system, and all SM components remain for all steps of the fusion assay (see Tables S1 and S2 and STAR Methods for more details). (B) Quantification of ISV-SM vesicle association with high (5:1) α-SNAP/NSF ratio (yellow) and low (2.5:1) α-SNAP/NSF ratio (red) Student’s t test p value = 0.2. (C) Fusion during association is largely suppressed at the higher (yellow) α-SNAP/NSF ratio. *p < 0.05, Student’s t test. (D) Fusion latency between ISV association and fusion. Ca^2+^-independent fusion shifts faster at the lower α-SNAP:NSF ratio (KS test p value <0.05). (E) Quantification of ISV dissociation events shows no difference between the α-SNAP/NSF ratios. Student’s t test showed no significant difference (p = 0.5). (F) Cumulative probability histogram of dwell times after a minimum association time of 4 s also shows no change in the dwell time in high or low α-SNAP/NSF molar ratios (KS test p value = 0.48). (G) Quantification of vesicles associated after the association period (solid bars) and after a 5-min incubation followed by washout (striped bars). In all cases, the number of pairs formed increases even after washout irrespective of the α-SNAP/NSF ratio or when the SM conversion and regeneration components are removed by washing (“α-SNAP & NSF removed” condition). Data from panel B is included for direct comparison. (H) Quantification of the total triggered fusion using high (yellow) and low (red) α-SNAP/NSF ratios compared to when the disassembly components were removed before triggering fusion (light gray). (I) Histogram of triggered fusion after Ca^2+^ injection using high α-SNAP/NSF (dashed line); black lines denote a sum of two exponential decay functions fit by least-squares minimization. Inset is the same population rescaled. (J) Histogram of triggered fusion at low α-SNAP/NSF ratio. The decay was best fit with a single exponential function. (K) Cumulative probability histogram of fusion events shows that lower α-SNAP/NSF ratio produces a distribution more tightly associated with Ca^2+^ arrival (KS test p value <0.05). In contrast, the kinetics of fusion is slowed at the higher α-SNAP/NSF molar ratio. (L) Ratio of fusion events in the first second (5 acquisition frames) after Ca^2+^ arrival to all fusion events (referred to as synchronization). All fusion data, both Ca^2+^-dependent and Ca^2+^-independent, are normalized to the total number of vesicle pairs. Data are presented as mean ± SEM for association, fusion during association, and dissociation measurements for several replicates (Tables S1 and S2). For all bar graphs containing more than two groups, a one-way ANOVA with post hoc Tukey-Kramer analysis was performed; *p < 0.05, **p < 0.005; n.s., not significant. For all cumulative probability histograms, a KS test was performed.

We used our ISV-SM system in the same configuration as the previous experiment but kept NSF, α-SNAP, Munc18, and the regeneration components present throughout all stages of the fusion assay (Figure 5A). After tethering SM vesicles to the imaging surface, we removed free SM vesicles using a wash solution that contained all conversion components. We then added ISVs in a solution containing all conversion components (supplemented with 1 μM complexin, 1 μM SNAP-25, and 5 μM MUN). We used this same buffer without ISVs as a wash buffer in all subsequent stages of the experiment; thus, all components were present throughout the entire experiment. We compared two ratios of α-SNAP to NSF: a higher 5:1 ratio that guarantees full α-SNAPoccupancyofthe20Sparticleaccordingto cryo-electron microscopy structures,^40^ and a lower ratio of 2.5:1 that has been used in previous *in vitro* liposome fusion assays.^41^

The ISV-SM association was similar for both α-SNAP concentrations (Figure 5B). However, Ca^2+^-independent fusion during association was α-SNAP concentration dependent (Figure 5C). Despite the increase in fusion during association, the kinetics of fusion was similar between the two concentrations of α-SNAP (Figures 5D and S4G). The dissociation and dwell times were also nearly identical (Figures 5E and 5F, respectively; Figure S4H). The ISV-SM association after washing was again increased and similar to when α-SNAP and NSF were removed (Figure 5G).

Surprisingly, we observed a profound decrease in total Ca^2+^-triggered fusion when all components were always present after initial SM vesicle formation (Figure 5H). Moreover, triggered fusion was more desynchronized at the higher α-SNAP concentration, with the fusion histogram being fit by the sum of two exponential decays with fast decay rate constant of 0.3 s^−1^ (contributing to 67% of the fit) and a slower decay rate constant of 0.078 s^−1^, while at the lower α-SNAP concentration the decay curve was best fit with a single exponential function with a decay constant of 0.6 s^−1^ (Figures 5I–5K). There was more fusion synchrony at the lower α-SNAP concentration (Figure 5L). While inhibition of fusion by α-SNAP is unsurprising, we expected MUN to be able to work with Munc18 to protect formed SNARE complexes as described previously.^5,41^

### Munc13 alleviates α-SNAP-dependent inhibition of triggered fusion

**Figure 6.**
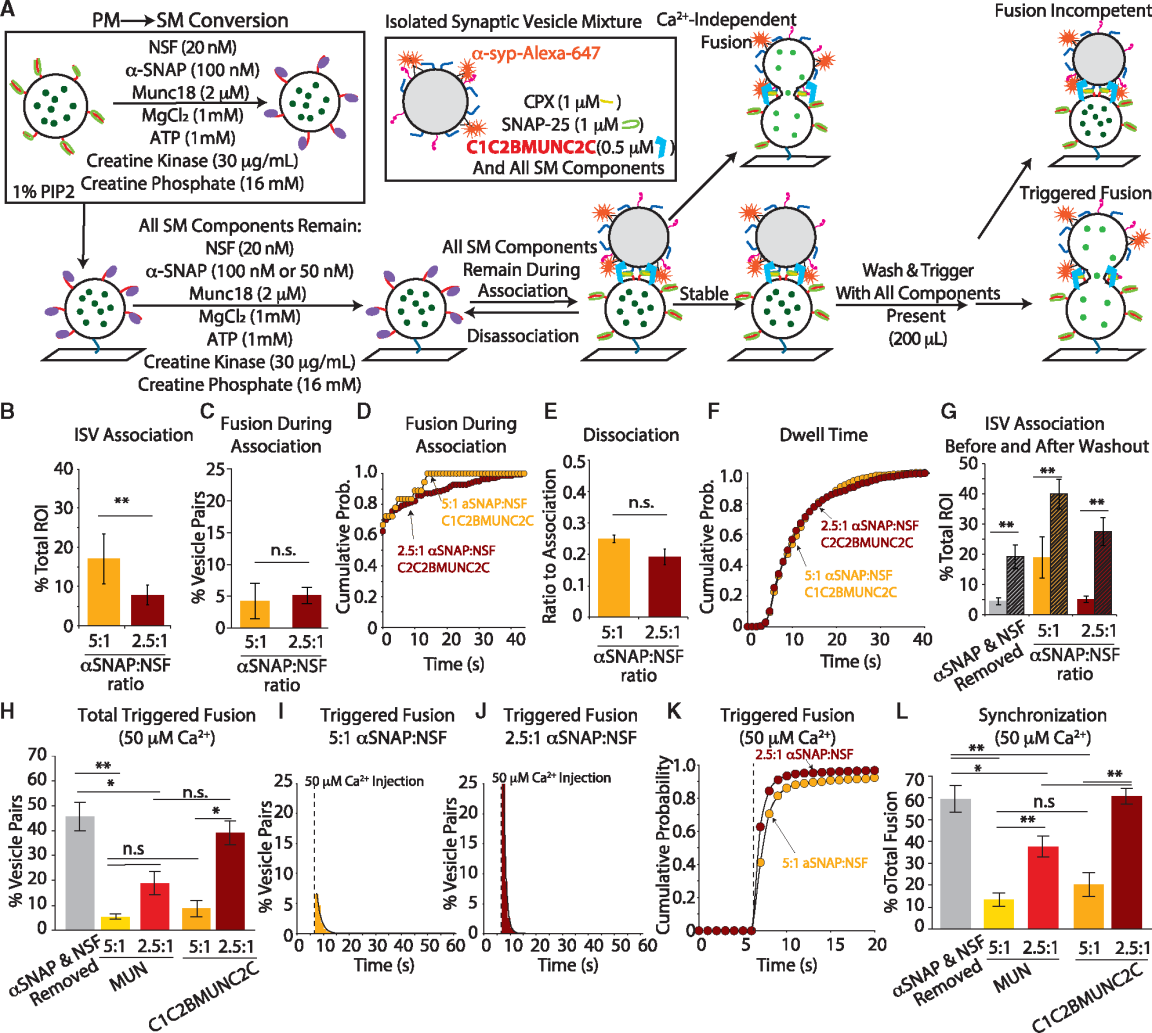
C1C2BMUNC2C rescues α-SNAP-dependent inhibition and desynchronization of fusion (A) Experimental scheme (changes to scheme in Figure 5A are indicated in bold). The C1C2BMUNC2C fragment instead of the MUN domain of Munc13 was used (see Tables S1 and S2 and STAR Methods for more details). (B) In the presence of C1C2BMUNC2C, ISV-SM association is elevated in high α-SNAP/NSF ratio (orange) compared to low α-SNAP/NSF ratio (dark red). (C) Ca^2+^-independent fusion during association in the presence of C1C2BMUNC2C is unaffected by α-SNAP concentration. (D) Cumulative probability histogram of fusion latency. Decreasing α-SNAP caused a shift toward longer fusion latency (KS test p value = 0.019). (E) In the presence of C1C2BMUNC2C, dissociation is unaffected by α-SNAP concentration. (F) Cumulative probability of histogram of dwell times after a minimum association time of 4 s shows no difference between α-SNAP concentrations (KS test p value = 0.9). (G) ISV-SM association after the association period (solid bars) and after washout (striped bars) shows an increase in association in all conditions. Note that there was no incubation period for the α-SNAP/NSF ratio conditions, while the “α-SNAP & NSF Removal” condition followed a 5-min incubation. Data from panel B are included for direct comparison. (H) C1C2BMUNC2C rescues Ca^2+^-dependent fusion in the low α-SNAP condition. (I) Histogram of Ca^2+^-triggered fusion in the presence of high (5:1) α-SNAP/NSF ratio. The black line is a single exponential decay function fit by least-squares minimization, with a decay rate constant of 0.6 s^−1^. (J) Histogram of Ca^2+^-triggered fusion in the presence of reduced (2.5:1) α-SNAP/NSF ratio. Again, the black line is a single exponential decay function fit by least-squares minimization, with a decay constant of 1.08 s^−1^. (K) Cumulative probability histogram of fusion kinetics. Again, at the lower α-SNAP concentration (dark red), fusion is more synchronized with Ca^2+^ arrival (KS test p value <0.05). (L) Ratio of fusion events in the first second (5 acquisition frames) after Ca^2+^ arrival to all fusion events (referred to as synchronization). All association data are normalized to the total number of SM vesicles (“total ROIs”). All fusion data, both Ca^2+^-dependent and Ca^2+^-independent, are normalized to the total number of vesicle pairs. Data are presented as mean ± SEM (Tables S1 and S2). For all bar graphs, a one-way ANOVA with post hoc Tukey-Kramer analysis was performed; *p < 0.05, **p < 0.005; n.s., not significant. For all cumulative probability histograms, a KS test was performed for all pairwise comparisons.

In the final experiments, we used a longer fragment of Munc13 (529–1,725 Δ1,408–1,452, referred to as C1C2BMUNC2C^5^) that includes the C1, C2B, and C2C domains in addition to the MUN domain (Figure 6A). Note that our C1C2BMUNC2C construct has a 6-residue C-terminal truncation compared to the recently identified Munc13 C-terminal (MCT) domain.^42^ We again tested the same two α-SNAP-to-NSF ratios.

During ISV-SM association, we observed an elevated ISVSM pair formation when the C1C2BMUNC2C construct was used at the higher α-SNAP concentration rather than the lower concentration (compare Figure 6B with Figure 5B). The α-SNAP concentration did not alter the Ca^2+^-independent fusion amount or kinetics in the presence of C1C2BMUNC2C (Figures 6C and 6D). Both dissociation and dwell times were independent of α-SNAP concentration (Figures 6E and 6F). However, we noticed that after 5-min incubation and washout, a substantial number of new associations had formed. Therefore, we did not perform the 5-min incubation after association and instead washed immediately. Even between stopping acquisition and washing, there was a substantial increase in ISV-SM vesicle association in the presence of C1C2BMUNC2C. This is unsurprising, given that Munc13 is expected to form a bridge between the ISV and SM vesicles via its C2C domain.^42–45^ After this wash step, we observed a comparable increase in the number of ISV-SM pairs remaining (Figure 6G).

For Ca^2+^-triggered fusion, in the presence of C1C2MUNC2C we again observed a reduction in total fusion at the higher α-SNAP concentration (Figure 6H). However, at the lower α-SNAP concentration, triggered fusion completely returned to levels observed when NSF and α-SNAP were removed altogether before Ca^2+^ triggering (Figure 6H). At the higher α-SNAP concentration, the kinetics of triggered fusion in the presence of C1C2BMUNC2C fit a single exponential decay with a decay rate constant of 0.6 s^−1^ with 20.0% ± 5.4% of fusion occurring in the first second of Ca^2+^ triggering (Figures 6I–6L). At the lower α-SNAP concentration, C1C2BMUNC2C returned fusion kinetics to fit a single exponential decay rate with a constant of 1.08 s^−1^, and 61.0% ± 3.6% of triggered fusion occurred in the first second after Ca^2+^ triggering (Figures 6J–6L). In the most complete reconstitution experiments, the fusion histograms do not contain a detectable slow-decay component. Taken together, we find that α-SNAP inhibits Ca^2+^-triggered fusion under some conditions and that C1C2BMUNC2C can alleviate this inhibition, whereas the MUN domain cannot.

## DISCUSSION

**Figure 7.**
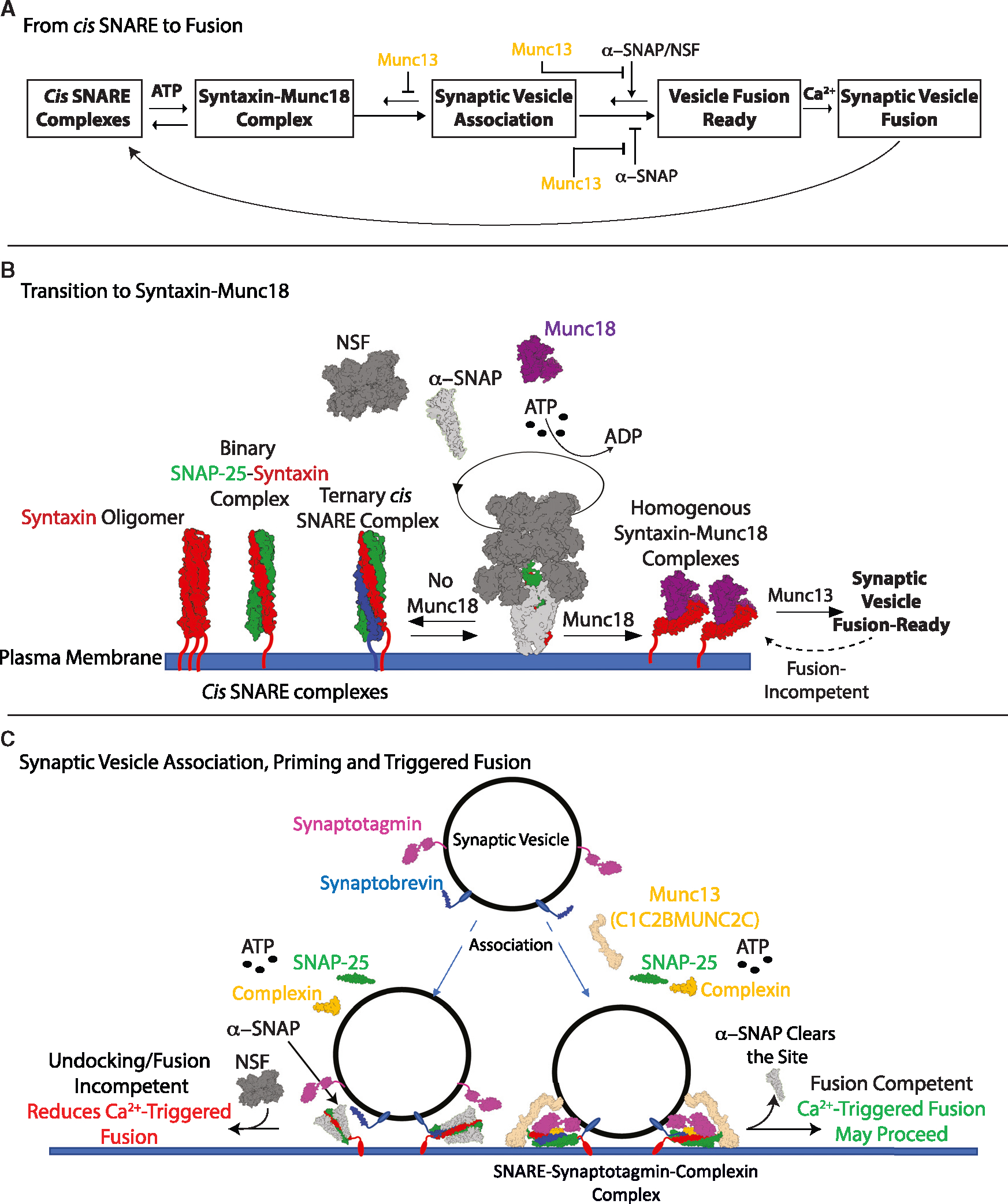
Model of how C1C2BMUNC2C enables clearance of α-SNAP from the synaptic vesicle interface (A) Ternary cis SNARE species are converted to syntaxin-Munc18 complexes via NSF-α-SNAP-mediated disassembly driven by ATP. Synaptic vesicles may then associate and move to a fusion-ready state, which may finally undergo Ca^2+^-dependent fusion. α-SNAP antagonizes the generation of fusion-ready synaptic vesicles. Munc13, and not just the MUN domain, in turn inhibits the actions of α-SNAP/NSF to promote fusion. (B) Before synaptic vesicle-plasma membrane association, syntaxin exists in a mixture of complexes, for example, tetrameric syntaxin oligomers (K.I. White, Y.A.Khan, J. Diao, K. Qiu, S.C.-C., R.A.P., L.E., and A.T.B., unpublished data), syntaxin-SNAP-25 binary, or ternary cis SNARE (post fusion) species. Repeated rounds of NSF-α-SNAP, mediated disassembly shift syntaxins toward a homogeneous syntaxin-Munc18 population as a starting point for synaptic vesicle arrival and formation of fusogenic trans SNARE complex by the catalytic action of Munc13. (C) Next, the primed SNARE-synaptotagmin-complexin complex forms,^7,8^ although the transition to this complex can be inhibited by α-SNAP. In the presence of MUN, α-SNAP remains present and inhibits vesicle fusion, while the longer C1C2BMUNC2C fragment of Munc13 may permit the clearance of α-SNAP and leads to the fusion-competent SNARE-synaptotagmin-complexin complex.

Classically, synaptic vesicle docking has been considered a relatively static state^46,47^ that precedes synaptic vesicle priming, also perhaps a static state, so that vesicles rest in a fusion-ready state^48^ (Figure 7A). Indeed, the ability to capture membrane-proximal synaptic vesicles in electron microscopy images of resting neurons supports this notion, but a growing body of evidence suggests that both vesicle docking and vesicle priming may be reversible.^18–20,49^ Rapid synaptic vesicle fusion with the plasma membrane in the neuron is a highly coordinated process involving several active zone proteins.^50,51^ Here, we propose a model wherein α-SNAP and NSF drive retrograde processes that antagonize a fusion-ready state, while Munc13—and specifically the C1C2BMUNC2C fragment and not the MUN domain alone—facilitates progression toward a fusion-ready vesicle state (Figure 7A). Together, these competing components produce a fault-tolerant and regulatable (and reversible) synaptic vesicle fusion cycle. We base our model on these insights. (1) ISV association with SM vesicles (a plasma membrane mimic) was reversible (10%–40%, Figures 2E, 3E, 4E, 5E, and 6E). (2) The PIP2 concentration in the plasma membrane regulated synaptic vesicle fusion and ISV-SM association (Figure 3). (3) Maintenance of constant ATP concentration resulted in less Ca^2+^-independent fusion during assocition, improved ISV association, stability, and synchronization of fusion upon Ca^2+^ triggering (Figure 4). Thus, both PIP2 and ATP concentrations may regulate synaptic vesicle fusion activity at the synapse.^33^ (4) When all components were present at all stages of our assay (i.e., also during triggered fusion—as expected to be the case in neurons), the dissociation rate reached a similar range (20%–30%, Figures 5E and 6E), but Ca^2+^-triggered fusion was reduced at the higher α-SNAP concentration when the catalytic MUN domain of Munc13 was used (Figures 5H and 5L). This inhibition by α-SNAP was alleviated by the longer C1C2BMUNC2C fragment of Munc13 (Figures 6H and 6L).

### Multiple rounds of NSF-α-SNAP-mediated disassembly

Previous studies have estimated that ternary SNARE complex disassembly consumes up to 50 ATP molecules per SNARE complex,^52^ although other experiments suggested that 12 ATP molecules suffice.^53^ In the axon terminal, the resting baseline ATP has been reported to be between 1 and 4 mM.^54,55^ Here, we used the lower end of this estimation, 1 mM ATP (in 200 μL of PM-vesicles), which should still be more than sufficient to disassemble ~6 × 10^15^ binary (syntaxin-1-SNAP-25) SNARE complexes, considering that binary SNARE disassembly likely requires less ATP.^56^ We estimate that we incorporate ~120 binary complexes per synthetic PM vesicle^57^; thus, the time and amount of ATP should have been sufficient to convert all binary SNARE complexes.

However, when the ATP-regeneration system was added, essentially maintaining the ATP concentration during the acquisition period, we observed an increase in ISV dissociation (Figure 4E). We also observed an increase in total ISV-SM association even when NSF and α-SNAP were removed by washing during association (Figure 4G). Together, failure to form a stable ternary *trans* SNARE complex likely results in a reversion to a closed syntaxin-1-Munc18 state, where ISV-SM vesicle association may again be attempted, consistent with previous reports.^58,59^ However, in the absence of the regeneration system, there exists the possibility of potential reformation of a cis SNARE complex, thus necessitating multiple rounds of disassembly (Figure 7B).

We observed increased triggered fusion synchrony in our experiments when the ATP-regeneration system was added (Figure 4M). We also observed a decrease in fusion during association (Figure 4C), again suggesting that multiple rounds of disassembly may be required to fully convert the syntaxin-1SNAP-25 binary complex population to syntaxin-1-Munc18 complexes. For example, there could be reassembly of the binary complex after disassembly or otherwise incompetent acceptor complexes, for instance, syntaxin-1 homo- and hetero-oligomers (K.I. White, Y.A. Khan, J. Diao, K. Qiu, S.C.-C., R.A.P., L.E., and A.T.B., unpublished data).^60–62^ Incorrect assembly of the SNARE complexes may result in fusion independent of Ca^2+^, fusion poorly synchronized to Ca^2+^ arrival, or complexes that are not fusogenic. In addition, the disassembly of *trans* SNARE complexes can occur under some conditions,^16^ effectively inhibiting fusion.

Taken together, when continual disassembly of SNARE complexes is allowed, ISV-SM vesicle association becomes ultimately more stable and, in turn, evoked fusion becomes more synchronous.

### C1C2BMUNC2C and not MUN alone alleviates α-SNAP-dependent inhibition of fusion

When we maintained Munc18, NSF, α-SNAP, complexin, synaptotagmin, and ATP throughout the fusion assay—as would be the case in neurons—we found a profound inhibition of triggered fusion in the presence of the MUN domain (Figure 5). This inhibition was α-SNAP concentration dependent (Figure 5H); in other words, the proofreading machinery appears to arrest fusion when only the MUN domain is present. Remarkably, we were able to alleviate inhibition of triggered fusion by the C1C2BMUNC2C fragment of Munc13 (including the C1, C2B, MUN, and C2C domains) (Figures 6H and 6L). This suggests that portions of Munc13 ancillary to the MUN domain make the system more permissive to fusion. We note that the MUN domain alone catalyzes the transit from the syntaxin-1-Munc18 complex to the ternary SNARE complex and is critical for transition to the ternary SNARE complex.^15,58,63^ However, mutations in these domains ancillary to MUN such as membrane-binding domains^45,64^ and Ca^2+^-binding domains (specifically the C1, C2B, and Ca^2+^∕calmodulin domains) all have similar phenotypes whereby basal synaptictransmission is unaffected but short-term facilitation is inhibited.^65–68^ Thus, α-SNAP can act as an antagonistic factor for Ca^2+^-triggered fusion that is alleviated by Munc13 (in conjunction with Munc18), consistent with previous findings in *munc13–1* null synapses that result in NSF-dependent depriming of synaptic vesicles.^21^

Our results suggest that the membrane bridging/tethering activity by the C1C2BMUNC2C fragment of Munc13^43,44^ physically permits the exchanging and clearance of α-SNAP from the vesicle-plasma membrane interface. In addition, the presence of the C1, C2B, and C2C domains effectively increases the local concentration of Munc13 near the synaptic vesicle docking site. The lateral conformation of C1C2BMUNC2C brings the synaptic vesicle closer to the plasma membrane (Figure 7C, right). This shorter membrane distance might sterically hinder α-SNAP from engaging the *trans* SNARE complex. Ca^2+^-induced conformational changes of C1C2BMUNC2C could also regulate this process.^42–44^ Moreover, Munc18 and Munc13 act together^5,41^ to set the stage for the efficient binding of synaptotagmins and complexins. In turn, complexin and synaptotagmin binding and formation of the primed SNARE-synaptotagmin-complexin complex^7,8^ compete with α-SNAP binding and, therefore, prevent NSF-α-SNAP-mediated disassembly.^15^ Finally, the domain 3a of Munc18 interacts with the full-length assembled SNARE complex,^69^ and Munc13 interacts directly with synaptobrevin and SNAP-25 during addition to the SNARE complex to ensure parallel SNARE complex formation.^6,59,70,71^ Thus, these processes promote the formation of primed fusogenic SNARE-synaptotagmin-complexin complexes^7,8^ and protect the *trans* SNARE complex from α-SNAP binding and disassembly. In contrast, improperly or incompletely formed SNARE complexes may not efficiently bind complexin and synaptotagmins, consequently enabling α-SNAP binding for subsequent disassembly by NSF and effectively inhibiting any fusion activity by these complexes.

### Limitations of the study

We still lack the components necessary for a complete physiologically faithful recreation of synaptic vesicle fusion. Alternative active zone proteins such as CAPS^72^ may substitute or augment Munc13 function; our reconstituted *ex vivo*/*in vitro* system provides a platform from which to examine their roles further. Therefore, careful consideration is required when drawing parallels between processes observed in our assay and *in situ* physiological processes. Moreover, fusion between two spherical vesicles also imperfectly mimics fusion with the larger and flatter plasma membrane. Thus, while it is tempting to connect our observations to the physiological process of priming, we acknowledge that some aspects of priming were not assessed here.

## STAR★METHODS

### RESOURCE AVAILABILITY

#### Lead contact

Additional information and requests for resources should be directed to the lead contact, Dr. Axel T. Brunger (brunger@stanford.edu).

#### Materials availability

All protein and peptide constructs have been deposited in Addgene.org.

#### Data and code availability

- All imaging data and Excel files for all bar charts, histograms, and distribution plots have been deposited in the Stanford Digital Repository at https://doi.org/10.25740/wc823yz5804
- The smCamera software (Taekjip Ha laboratory, Johns Hopkins University, Baltimore) was used for acquisition. MATLAB and Excel were used to generate all curves and graphs. MATLAB scripts were used for the analysis of the single-vesicle fusion experiments. Custom MATLAB scripts (Fusion_Detector_2_0_7_JL_atb_2.m) are available at https://github.com/brungerlab/single_molecule_matlab_scripts
- Any additional information required to reanalyze the data reported in this work paper is available from the lead contact upon request.

### EXPERIMENTAL MODEL AND STUDY PARTICIPANT DETAILS

P20 Wild-type CD1 (IMSR_CRL:022) *Mus musculus* of mixed gender were kept with food and water *ad libitum* until sacrificed. All procedures involving the use of animals were performed in accordance with the guidelines of the National Institutes of Health and were approved by the Institutional Animal Care and Use Committee (IACUC, protocol #29981)

### METHOD DETAILS

#### LP2 preparation, vGlut peptide preparation, and isolation of synaptic vesicles

Vesicles were isolated similarly to a previous protocol^73^ with extensions (Figure S1 and ref; ^26^). Briefly, 6–12 wild-type CD1 mixedgender mice were sacrificed at P20. Mouse brains were pooled and homogenized in a Dounce homogenizer 3 times with the “A” pestle and 3 times with the B pestle. The homogenate was then spun in a JA-20 rotor at 2,700 rpm (880x g) for 10 min. The supernatant was transferred to a new tube and spun at 10,000 rpm (12,064× g) for 15 min. The resulting supernatant was removed, and the pellet was collected, transferred to a new tube, and spun again at 11,000 rpm (14,597× g) for 15 min. Synaptosomes were then collected from this pellet and hypo-osmotically lysed by the addition of ultrapure H_2_O (9:1). This solution was then spun at 19,500 rpm (45,871× g) for 20 min. The supernatant was removed and ultracentrifuged using a Ti-70 rotor at 50,000 rpm (256,631× g) for 2 h. The resulting pellet containing synaptic vesicles was then homogenized in 2 mL of PBS, and vesicles were mechanically sheered through a 27-gauge needle. Total protein concentration was determined by BCA, aliquoted, flash frozen in liquid nitrogen, and stored at −80°C until use.

VGLUT peptide (GPPGISGGGG GILGSDESEM EDEAEPPGAP PAPPPSYGAT HSTVQPPRPP PPVRDY) was expressed using *Escherichia coli* BL21 (DE3) cells (Invitrogen) and purified as described in ref. ^26^. vGlut peptide was concentrated to 10–15 mg/mL.

LP2 was thawed on ice and diluted to 500 μg/mL in vesicle buffer (20 mM HEPES pH 7.4, 90 mM NaCl, 20 μM EGTA), incubated with 5 μL of monoclonal mouse anti-vGlut antibody (Synaptic Systems) overnight at 4°C. 50 μL of Protein G paramagnetic beads, Dynabeads (Thermo Fisher Scientific), were washed 3x in PBS containing 0.5% BSA. The labeled LP2 mixture was then added to the Dynabeads and incubated for 2 h. Beads were then separated using a magnet, and the flow-through was removed. Beads were washed once in PBS containing 0.5% BSA and twice in PBS alone. ISVs were then eluted 3x by adding 25 μL of 5 mg/mL vGlut peptide. Beads were incubated in vGlut peptide for 20 min between elutions.

ISVs were fluorescently labeled by incubation in 1:1000 polyclonal anti-synaptophysin Alexa Fluor 647 antibody (abcam) overnight. Vesicles were then dialyzed in “vesicle buffer” (20 μM HEPES pH 7.4, 90 mM NaCl, 20 mM EGTA) using a 300 kDa cutoff dialysis cassette FloatALyzer (Spectra-Por) for 2 h. Vesicles were then immediately used in the fusion assay or for electron microscopy.

#### Preparation of purified Munc13, Munc18, syntaxin-1A, SNAP-25A, complexin-1, NSF, α-SNAP

We used the same constructs and protocols to purify Munc13 (MUN and C1C2BMUNC2C), Munc18, syntaxin-1, SNAP-25A, complexin-1, NSF and α-SNAP as described previously.^6^ Briefly, we summarize the protocols below:

The MUN domain (amino acid range 859–1531, excluding residues 1408–1452) was cloned into an N-terminal hexahistidine TEV cleavable construct and was expressed in BL21 (DE3) *E. coli*. Cells were collected by centrifugation lysed by sonication using a Sonicator ultrasonic processor XL (Misonix Corporation). The cell lysate was then centrifuged, and the supernatant was applied to a 5 mL bed volume of Ni-NTA-agarose column (Qiagen). The column was washed with the lysis buffer, and then the protein was eluted using 50 mM Tris pH 8.5, 300 mM NaCl, 400 mM imidazole, 0.5 mM DTT (Dithiothreitol), and 10% glycerol. The elution was then collected into a 10 kDa MWCO SnakeSkin Dialysis Tubing (Thermo Scientific), TEV protease was added, and placed in dialysis buffer to remove the tag. The cleaved protein solution was applied to a Mono Q 4.6/100 ion exchange column (GE Healthcare). The protein was eluted using a linear gradient from 50 mM to 1 M NaCl over 30 column volumes. The appropriate fractions were pooled and applied to a HiLoad Superdex 200 16/60 PG size exclusion column (GE Healthcare) equilibrated in 20 mM Tris pH 8.5, 150 mM NaCl, 10% glycerol, and 5 mM DTT (Superdex buffer). The protein-containing fractions were pooled and concentrated, the protein concentration was measured by UV absorption at 280 nm, and aliquots were flash-frozen in liquid nitrogen and stored at −80°C.

The C1C2BMUNC2C fragment (amino acid range 529–1735, excluding residues 1408–1452) was cloned into a pFASTBAC vector with an N-terminal GST tag and a TEV cleavage site, as described in.^74^ Cells were collected and were lysed via 3 passes through the Avestin C5 homogenizer at 15000 psi. The lysate was clarified by centrifugation, the supernatant was mixed with 10 mL of glutathione Sepharose 4B (GE Healthcare). The beads were washed using an Akta Start system (GE Healthcare) and then eluted with 20 mM reduced glutathione. Peak fractions were pooled, and TEV protease was added to remove the GST. The protein was concentrated using a 50 kDa Molecular Weight cutoff filter (Millipore) and injected on a Superdex 200 16/60 column (GE Healthcare). Peak fractions were combined, and the protein concentration was measured by UV absorption at 280 nm. Aliquots of 200 mL were flash-frozen in liquid N2 and stored at −80°C.

Munc18a was expressed in BL21(DE3) cells from a pPROExHTa vector (Invitrogen) as an N-terminal hexahistidine tag with a tobacco etch virus (TEV) protease cleavage site to remove the tag. Cells were collected and lysed by three passes through the Emulsiflex C5 homogenizer (Avestin) at 15000 psi. Lysate was clarified by centrifugation and bound to 5 mL of Nickel NTA beads (Qiagen) and poured into a column, attached to an Akta Start System (GE Healthcare). Munc18a was eluted with 300 mM imidazole. Protein-containing fractions were combined, the tag was cleaved by TEV protease, and the mixture was dialyzed before being injected into a Mono Q column (GE Healthcare). The column was eluted using a linear NaCl gradient from 50 to 500 mM NaCl over 30 column volumes. Protein-containing fractions were combined and dialyzed against 1 L of 20 mM HEPES pH 7.5, 180 mM NaCl, 20 μM EGTA, and 0.1% 2-mercaptoethanol. The protein concentration was measured by absorption at 280 nm, and aliquots were flash-frozen in liquid nitrogen and stored at −80°C.

Full-length syntaxin-1A was expressed in *E. coli* C43 with a N-terminal, TEV protease-cleavable, hexa-histidine tag from plasmid pJEXPRESS414 at 25°C overnight in TB autoinducing media. Cell pellets from 6 L of culture were resuspeded in 500 mL of 1X phosphate-buffered saline, 1 mM Magnesium Sulfate, benzonase and 5 mM EGTA supplemented with Complete Protease Inhibitor Coctail tables (Roche). Cells were lysed by sonication (3 seconds on, 9 seconds off, 5 mins at 80% power) and passage through a cell disruptor (2 passes at 25,000 psi). Inclusion bodies were removed by two consecutive 11 minute centrifugation spins (11,000 rpm JA14 rotor (Beckman Coulter)), and the membrane fraction collected by centrifugation at 44,000 rpm for 1.75 hours in a (Ti-45 rotor) (Beckman Coulter). The membranes were further washed with buffer containing 10 mM Tris-HCl pH 7.5, 10 mM EDTA, 10% glycerol (w/v), and centrifuged at 43,000 rpm for 1 hour in a Ti-45 rotor. Membrane pellets were resuspended to a concentration of 5 mg of protein per mL in 20 mM HEPES pH 7.5, 500 mM NaCl, 1 mM TCEP, 10 mM imidazole, 10% glycerol (w/v), 1 mM PMSF and EDTA-free Complete Protease Inhibitor Cocktail. Dodecylmaltoside (Anatrace) was added to 2%, the mixture was sonicated in a water bath for 2 minutes and solubilized overnight at 4°C. The sample was centrifuged for 35 min at 42,000 rpm Ti-45 rotor and the supernatant was mixed with 1.5 mL of Nickel-NTA agarose (Qiagen) and incubated 2 hours at 4°C. After incubation the Ni beads were poured into a column and washed with 20 mM HEPES pH 7.5, 300 mM NaCl, 1 mM TCEP, 25 mM imidazole, 110 mM n-Octyl-β-D-Glucoside (OG; Anatrace) and 10% glycerol (w/v) and proteins eluted in the same buffer containing 450 mM imidazole and 1 M NaCl. 1 mM EDTA was immediately added to each fraction. Fractions were pooled and digested with 100 μg TEV protease for 30 min at room temperature, after which the TEV protease had precipitated. TEV was removed by centrifugation at 5,000 rpm for 10 min. The sample in the supernatant was subjected to a second round of TEV protease digestion overnight at 4°C. TEV was removed by centrifugation at 5,000 rpm for 10 minutes. Syntaxin was concentrated to 2 mL using a 10 kDa MWCO concentrator and loaded onto a Superdex 200 10/300 Increase (GE HEalthcare, Uppsala, Sweden) equilibrated in 20 mM HEPES pH 7.5, 300 mM NaCl, 1 mM TCEP, 110 mM OG and 10% glycerol (w/v). Fractions containing syntaxin were flash frozen and stored at −80°C until use.

Cysteine-free SNAP-25A was expressed with an N-terminal TEV protease cleavable hexahistidine tag from plasmid pTEV5 in *Escherichia coli* BL21(DE3). Cells were lysed by sonication, and the lysate was clarified by multiple rounds of centrifugation. The supernatant was bound to 5 mL of Nickel NTA resin (Qiagen) and eluted with 350 mM Imidazole. Protein-containing fractions were combined, DTT and EDTA were added to 1 mM, and TEV protease was added to remove the hexahistidine tag. SNAP-25 was concentrated in a 15 mL Amicon Ultra centrifugal concentrator with a 10 kDa molecular weight cutoff membrane (Millipore) and injected on a Superdex 200 (16/60) column (GE Healthcare) or an S75 column (16/60 GE Healthcare). Protein-containing fractions were combined, the concentration of SNAP-25 was measured by absorbance at 280nm, and aliquots were frozen in liquid nitrogen.

Full-length wild-type complexin-1 (Cpx) was expressed in *E. coli* using BL21 (DE3) cells with a thrombin-cleavable N-terminal hexahistidine tag from plasmid pET28a (Novagen, EMD Chemicals). Cells were lysed by passing them through the Emulsiflex C5 homogenizer at 15,000 psi three times. Lysate was clarified by centrifugation, and the supernatant was bound to a 4 mL bed volume of Nickel-NTA beads poured into a column, attached to an Akta start, and eluted with 450 imidazole. Protein-containing fractions were combined, and thrombin (Haematologic Technologies, Essex Junction) was added. The elution was injected onto a Mono Q (5/50) column eluted with a linear 50 mM to 500 mM NaCl gradient. Protein-containing fractions were combined and dialyzed against 20 mM HEPES pH 7.5, 100 mM NaCl, and 4 mM DTT overnight at 4°C, followed by concentration using a 3 kDa cutoff dialysis cassette. The concentration was measured by absorbance at 280 nm, and aliquots were frozen in liquid nitrogen.

Chinese hamster NSF with a tobacco etch virus (TEV) protease cleavable N-terminal His-tag was expressed from pPROEX-1 vector in *E. coli* BL21(DE3)-RIL cells (Agilent Technologies) at 25°C overnight using autoinducing LB medium. After collecting the cells by centrifugation, the pellet was resuspended in lysis buffer (50 mM Tris-HCl pH 8.0, 300 mM NaCl, 50 mM imidazole, and 0.5 mM TCEP), and were subjected to sonication and centrifugation. The cleared lysate was loaded onto a HisTrap column (GE Healthcare), and washed with lysis buffer. NSF was eluted using elution buffer (lysis buffer supplemented with 350 mM imidazole). The fresh elution was pooled, concentrated, and supplemented with final concentrations of 1 mM EDTA, 1 mM ATP, and 10% glycerol immediately to prevent aggregation and precipitation. The concentrated protein was immediately loaded onto a Superdex 200 16/60 column (GE Healthcare) that was pre-equilibrated with SEC Buffer (50 mM Tris-HCl pH 8.0, 150 mM NaCl, 1 mM EDTA, 1 mM ATP, 1 mM DTT, and 10% glycerol)

Rat α-SNAP was expressed with an N-terminal TEV cleavable decahistidine tag from a codon-optimized plasmid using the pJexpress414 backbone (DNA 2.0) in *E.coli* BL21 (DE3). The clarified lysate was loaded onto a 5 mL Ni-NTA agarose column, washed, and eluted in 50 mM Tris pH 8.0, 300 mM NaCl, supplemented with 60 mM imidazole and 350 mM imidazole, respectively. TEV protease was added to pooled fractions; the protein was then run through a Superdex 200 16/600 size exclusion column. The protein-containing fractions were pooled and concentrated, the protein concentration was measured by UV absorption at 280 nm, and aliquots were flash-frozen and stored at −80°C.

#### PM vesicle generation

PM vesicles were generated as described previously,^6^ except that PM vesicles were formed in the presence of sulforhodamine B (ThermoFisher). Briefly, a lipid film of Porcine Total Brain Extract (Avanti Polar Lipids), 1% or 3% PI(4,5)P2 (Avanti Polar Lipids), 1% Biotin PE, and 1% DAG (see STAR Methods Key Resources Table for specific Lipid information) was generated by combining the lipids followed by evaporation under argon. In the case of vesicles containing 1% PIP2, equimolar POPC was used to replace the missing PIP2. Lipid films were allowed to dry overnight under a vacuum. Vesicles were reconstituted by adding syntaxin-1 and SNAP-25 in vesicle buffer supplemented with 5 mg/mL of sulforhodamine B. The sulforhodamine concentration was optimized to achieve a large fluorescence dequenching effect upon content mixing (fusion) while minimizing possible effects on protein reconstitution. Lipids were then added to a Sepharose CL-4B column (Millipore Sigma) to separate vesicles from the unincorporated dye.

#### PM to SM vesicle conversion

In contrast to our previously reported SM vesicle conversion method,^6^ here we performed the PM-to-SM conversion in solution before tethering vesicles to the imaging surface (Figure 1A). PM vesicles were incubated in a PM-to-SM conversion mixture containing 0.02 μM NSF, 0.1 μM α-SNAP, 2 μM Munc18, 1000 μM ATP, 1000 μM MgCl_2_, 500 mM TCEP, for 30 min. The concentrations of NSF and α-SNAP are within estimated physiological concentrations.^75^ SM vesicles were added to the imaging surface for 15 min, followed by washout with vesicle buffer supplemented with 2 μM Munc18 and 500 μM TCEP. Before injection, we adjusted the labeled ISVcontaining solution to include a final concentration of 1 mM complexin, 1 μM SNAP-25, 500 μM TCEP, and either 5 μM or no MUN.

#### Fusion assay data collection

Quartz slides were prepared as described previously.^6^ Briefly, slides were previously functionalized by coating with PEG/biotin-PEG at 0.01% and kept at −20°C until use. Neutravidin (ThermoFisher) was added at 0.1 mg/mL and incubated for 5 min. Unbound neutravidin was removed by washing ~50 volumes of vesicle buffer. SM or PM vesicles were then added and incubated for 5 min (Figure 1A). Following incubation, unbound SM or PM vesicles were removed by ~50 volumes of vesicle buffer containing components described in the experiments. Slides were then mounted to the microscope in preparation for imaging of vesicle association. A 10x Munc13 solution in standard vesicle buffer was added to the ISVs before addition to the slide such that the final concentrations of proteins were 1 μM complexin, 1 μM SNAP-25, 500 μM TCEP. The concentrations of MUN and C1C2BMUNC2C are described in the experiment. Labeled ISVs were then added in vesicle buffer to the slide with the tethered SM vesicles, and the ISV-SM vesicle association was monitored for 1 min at ~5 Hz frame acquisition. After association, unbound ISVs were again washed away with ~50 volumes of vesicle buffer. Where denoted, ISVs were allowed to associate for 5 min. After incubation, SM-ISV samples were washed with ~50 volumes of vesicle buffer supplemented with components depending on the specific experiment (Figures 1A, 3A, 4A, and 5A). For measuring Ca^2+^-independent fusion, vesicle fusion was monitored again for 1 min after the 5-min incubation. Any fusion that occurred with the arrival of a new ISV was ignored, and only fusion between pre-existing pairs was measured. For triggering, we injected 50 μM Ca^2+^ or Mg^2+^ in a vesicle buffer supplemented with components described in the specific experiment and 100 ng of Alexa-647 NHS ester (Thermo Fisher Scientific). Vesicles were imaged for 6 s (30 frames) before Ca^2+^ injection (Figures S2 and S3). For the ISV-PM system, the same general method applies except that ISVs are added in buffer alone with no additional proteins. Similarly, wash buffer and fusion triggering buffers did not contain any additional proteins (i.e., no Munc13 and no complexin).

Prism-based total internal reflectance microscopy (TIRF) was performed on a Nikon Ecipse Ti-U with a 60x water immersion objective. Images were collected on an Andor iXon Ultra-888 EMCCD camera (Andor) attached to the microscope via a custom Optosplit II (Technical Instruments). The emission image was split into red (for monitoring the Alexa-647 fluorescence intensity) and green (for monitoring the sulforhodamine B fluorescence intensity) channels using a 594 nm Laser BrightLine Single-Edge Super-Resolution Laser Dichroic Beamsplitter (25.2 × 35.6 × 3.00 mm) (Semrock). The remaining optics are identical to those used previously in our lab.^6,76^ Images were collected using the smCamera software (Taekjip Ha laboratory, Johns Hopkins University, Baltimore). Regions of interest (ROIs) were selected as 3-pixel radius circles with peak pixel intensity 0.5% greater than the background using the smCamera Movie Analysis tool. The fluorescence intensities of ROIs were normally distributed, indicating that likely single SM or PM vesicles were attached to the imaging surface. Paired vesicles were determined by counting overlapping ROIs with an allowed error of 2 pixels using the smCamera Movie Mapper tool with a predetermined alignment file. The alignment file was generated using the same Movie Mapper tool of a movie of TetraSpeck microspheres (Thermo Fisher Scientific). Alignment files were regularly updated.

ISV-SM/PM vesicle association was detected by monitoring a stepwise increase in the “red” channel that monitors the Alexa-647 fluorescence intensity, while any fusion event that may occur during association and after triggering was detected in the green channel that monitors the sulforhodamine fluorescence intensity (Figures 1B–1F, S2A–S2G, S3A, and S3B).

#### Fusion assay data analysis

Analysis was performed in MATLAB (Mathworks) using custom fusion-detection scripts. For both the red (for monitoring the Alexa647 fluorescence intensity) and the green (for monitoring the sulforhodamine B fluorescence intensity) channels, each trace was corrected for baseline fluorescence intensity by averaging the first 4.6 s (23 acquisition time bins) and subtracting each time point by this baseline. Next, a moving average and standard deviation were generated consisting of 10 acquisition time bins to create a “prior” baseline value. A moving “hit” average of 8 acquisition time bins was then compared to the prior value. Any test value was considered a putative “hit” if its value was at least 1.4x greater than the prior baseline plus one standard deviation of the prior. Additionally, the average fluorescence intensity over 20 acquisition frames after the putative “hit,” as well as the final acquisition time bins of the entire trace, must also be minimally 1.4x greater than the beginning of the experiment. These two constraints ensured the collection of stepwise increases in fluorescence intensity and eliminated events where SM or PM vesicles might rupture, photobleach, or altogether disappear. Our custom software compiled a list of putative hits which were then manually inspected as true hits or false positives. We intentionally made these constraints relatively loose to ensure more true positives were counted than false negatives.

For Ca^2+^-independent fusion analysis, a hit in the red channel must coincide with or precede the increase in the green channel. Any change in the green channel without a change in the red channel was not counted. Additionally, any traces that included more than one increase in the red channel were counted as “multiple association events”, but any fusion occurring therein was not counted (Figure S2C). Association events were normalized to the total number of available SM or PM vesicles (labeled as total ROIs).

For detecting dissociation events (Figures 2F, 3E, 4F, 5F, 6F, and S2E), the fluorescence intensity in the red channel must abruptly decrease (as opposed to a sloping decrease). A decrease in fluorescence intensity was detected exactly as described above, except the value must instead be lower than the prior fluorescence minus one standard deviation of the prior fluorescence intensity. Note that this does limit the minimum dwell time to ~4 s so that a prior baseline can be established. Dissociation events and dwell times were normalized to the total number of available SM or PM vesicles (labeled as total ROIs)

In the case of triggered fusion experiments, the green channel was treated exactly as above. At the same time, the red fluorescence intensity values were not corrected for background fluorescence (doing so would correct out, and thus eliminate, the global increase in fluorescence), averaged together, and the first point that was 1.4x greater than the initial acquisition time bins plus one standard deviation of the initial acquisition time bins was determined to be the time of Ca^2+^ arrival (Figure S3). Fusion times were then corrected for the arrival of Ca^2+^ for each experiment. Fusion events were normalized to the total number of vesicle pairs formed before fusion. Note that for the PM vesicle system (Figures S2, and S3), ancillary proteins (i.e., complexin) were not included.

Movies were excluded from analysis if there was noticeable drift, errors in injection, or less than 1% of available SM or PM vesicles formed pairs (indicative of an injection error). Additionally, films were excluded from synchronization analysis if they did not contain more than five fusion events.

#### Transmission electron microscopy

Negative stain transmission electron microscopy was performed using Copper-Formvar 300 mesh grids (FCF-300 and CF-300 Electron Microscopy Sciences). Grids were glow discharged in argon gas for 20 s. 4 μL of ISV sample was then added and allowed to incubate on the grid for 1 min. Grids were then washed three times with ultra-pure water. The grid was then negatively stained using 1% uranyl acetate for 2 min, then blotted and dried at room temperature for ~20 min. For immunogold, sample was applied similarly, grids were then incubated in blocking buffer (0.5% BSA, 0.5% ovalbumin in PBS) for 1 h at 4°C. Grids were then washed three times for 5-min in PBST. Then incubated with 1:10 10 nm gold goat anti-rabbit secondary antibody (Electron Microscopy Sciences). Grids were again washed three times for 5-min in PBST then fixed in 8% glutaraldehyde for 30 s. For cryo-electron microscopy samples, graphene suspended monolayer on quantifoil gold R2/4 grids were pre-cleaned with hydrogen gas plasma generated by Fischione 1020 plasma cleaner (70% Power, 22 sccm) 40 s. Grids are then placed in a custom humidity chamber and 4 μL ISV sample is added and incubated for 5 min. Grids are then loaded onto a vitrobot (ThermoFisher), blotted and plunged into liquid ethane. Grids were stored in liquid nitrogen until imaging. All imaging was performed on a JOEL 1400 TEM with Gatan OneView sCMOS camera with drift correction. Images were analyzed in ImageJ.

### QUANTIFICATION AND STATISTICAL ANALYSIS

The fusion experiments were conducted with at least three different protein preparations and vesicle reconstitutions (details in figure captions and Table S1), and properties were calculated as the mean ± SEM of the technical replicates (i.e., movies). One-way ANOVA with post-hoc Tukey-Kramer analysis was used to test statistical significance in Figures 2–6 and S3 for the specified reference experiment. When only two groups were present, the unpaired Student’s t test was performed (data presented in Figures 5, 6B, 6C, and 6E). A paired Student’s t test was performed for paired comparisons presented in ISV before and after washout. For all histograms, distributions were fit by a sum of exponential decay functions using least squares minimization. To compare all distributions (i.e., fusion during association latency, dwell time, and triggered fusion), a KS-test was performed for all pairwise comparisons.

## Supplementary Material

1

## Figures and Tables

**KEY RESOURCES TABLE:** 

REAGENT or RESOURCE	SOURCE	IDENTIFIER

Antibodies		

vGlut	Synaptic Systems	Cat# 135-311
Synaptophysin Alexa 647	AbCam	Cat# ab196166
Goat-*anti*-Rabbit IgG 10 nm Gold	Electron Microscopy Sciences	Cat# 25128

Chemicals, peptides, and recombinant proteins		

Alexa 647 NHS Ester	ThermoFisher Scientific	Cat# A20006
Brain Total Lipid Extract	Avanti Polar Lipid	Cat# 131101C
1-Palmitoyl-2-oleoyl-sn-glycero-3-phosphocholine (POPC)	Avanti Polar Lipid	Cat# 850457C
L-α-phosphatidylinositol-4,5-bisphosphate (PIP2)	Avanti Polar Lipid	Cat# 840046X
1–2-dioleoyl-*sn*-glycerol (DAG)	Avanti Polar Lipid	Cat# 800811
1-Oleoyl-2-(12-biotinyl(aminododecanoyl))-*sn*-glycero-3-phosphoethanolamine (Biotin-PE)	Avanti Polar Lipid	Cat#860562C
mPEG-SVA	Laysan Bio	Cat#mPEG-SVA-5000
Biotin-PEG-SVA-5000	Laysan Bio	Cat# Biotin-PEG-SVA-5000
Sulforhodamine B	Invitrogen	Cat#S1307
Neutravidin	ThermoFisher Scientific	Cat# 31000
Dynabeads Protein G	Thermo Fisher Scientific	Cat#10004D
vGlut peptide (GPPGISGGGG GILGSDESEM EDEAEPPGAP PAPPPSYGAT HSTVQPPRPP PPVRDY)	Addgene.org	218677
Syntaxin1 (Stx1a Rat) pDC216	Addgene.org	115791
Synaptobrevin2 (Vamp2 Rat) pDC191	Addgene.org	115792
SNAP25 (Snap25 Rat) PDC158	Addgene.org	115793
NSF (NSF Chinese Hamster)	Addgene.org	102239
αSNAP (αSNAP Rat)	Addgene.org	218673

Deposited data		

Imaging data and electron micrographs	Stanford Digital Repository	https://doi.org/10.25740/wc823yz5804

Experimental models: Cell lines		

*E.coli*. BL21 (DE3)	Invitrogen	Cat#C600003

Experimental models: Organisms/strains		

CD1 wild-type mice	Charles River	IMSR_CRL:022

Software and algorithms		

MATLAB	Mathworks	
Additional custom MATLAB scripts	This work (and Leitz J. et al. 2024)^26^	https://github.com/brungerlab/single_molecule_matlab_scripts
smCamera2	Taekjip Ha	http://ha.med.jhmi.edu/resources/#1464200861600-0fad9996-bfd4
ImageJ	Rasband, W.S., ImageJ, U.S. National Institutes of Health, Bethesda, Maryland, USA, 1997–2018	https://imagej.net/ij/

## Highlights

- A hybrid fusion assay that uses isolated synaptic vesicles and maintains presynaptic proteins
- NSF, α-SNAP, ATP, Munc18, and Munc13 perform quality control during vesicle association
- Continuous ATP-hydrolysis in NSF results in the most synchronous Ca^2+^-triggered fusion
- Domains ancillary to the MUN domain of Munc13 alleviate α-SNAP-dependent inhibition of fusion
