# Supplementary material for: Beyond the MUN domain, Munc13 controls priming and depriming of synaptic vesicles
Source: Cell Rep. Author manuscript; Available in PMC 2024 Jul 30. (PMC11286359; doi:10.1016/j.celrep.2024.114026)
Supplement: 1 [file NIHMS2000393-supplement-1.pdf]

Cell Reports, Volume *43*

Supplemental information

Beyond the MUN domain, Munc13 controls priming and depriming of synaptic vesicles

Jeremy Leitz, Chuchu Wang, Luis Esquivies, Richard A. Pfuetzner, John Jacob Peters, Sergio Couoh-Cardel, Austin L. Wang, and Axel T. Brunger

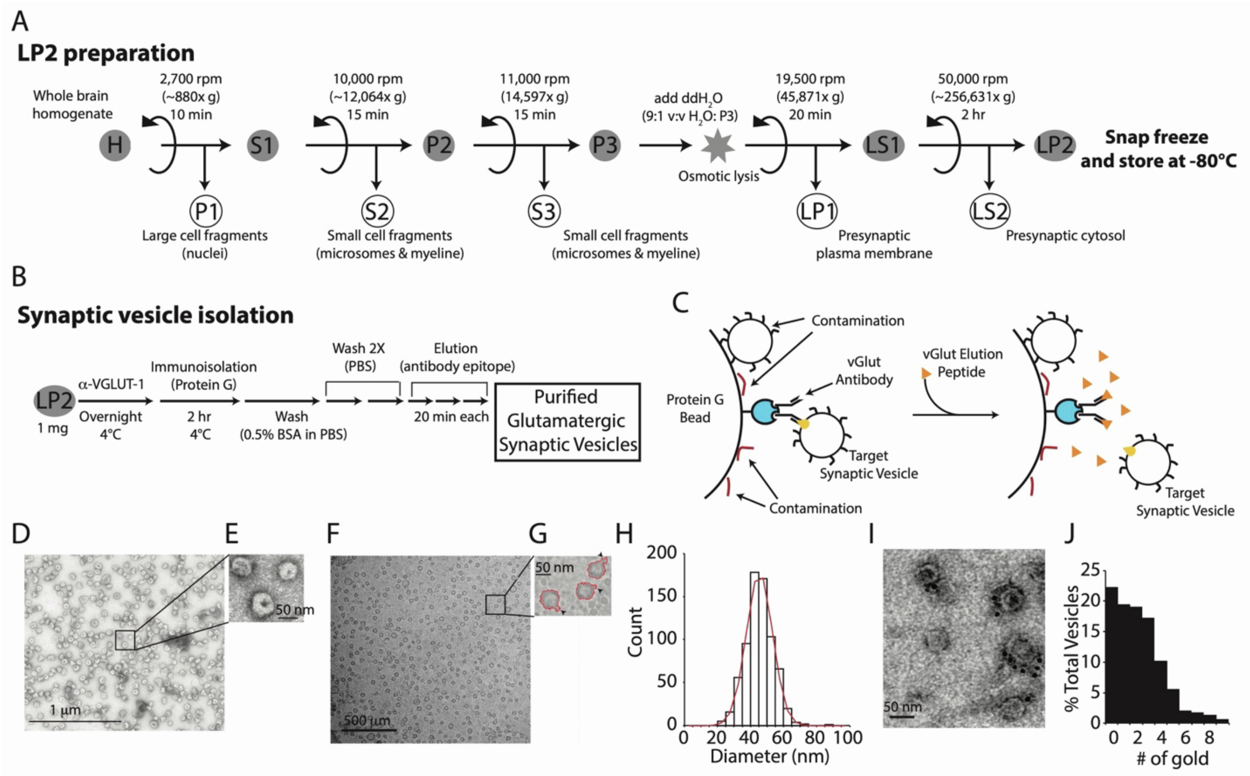

Figure S1. Isolation scheme of synaptic vesicles, related to STAR Methods. (**A**) Differential centrifugation scheme to prepare crude second lysis pellet (LP2) containing synaptic vesicles. (**B**) Isolation of synaptic vesicle from LP2. (**C**) Schematic of ISV elution using vGlut elution peptide. (**D** and **E**) Example negative stain electron micrographs from a typical isolation. (**F** and **G**) Example cryoEM micrographs of synaptic vesicle preparation. Red lines are contrast-dependent outlines generated in the program ImageJ (National Institutes of Health, Bethesda, MD) and used for diameter estimates based on circumference. Note the appearance of putative v-ATPase V1 domains protruding from vesicles (arrowheads). Gold fiducial markers were removed in ImageJ. (**H**) Vesicles measured in cryoEM micrographs had a mean diameter of 42.8 ± 7.9 nm (from 717 vesicles collected from 2 grids), as expected. (**I**) Example immunogold negative stain electron micrograph using an anti-synaptophysin antibody conjugated with 15nm gold. (**J**) Quantification of immunogold negative stain electron micrograph. More than 75% of vesicles were labeled by at least one antibody (in this particular preparation, from 283 vesicles collected from 12 grids). We observed only very few instances of unbound gold secondary antibody, suggesting that actual primary labeling may be slightly higher.

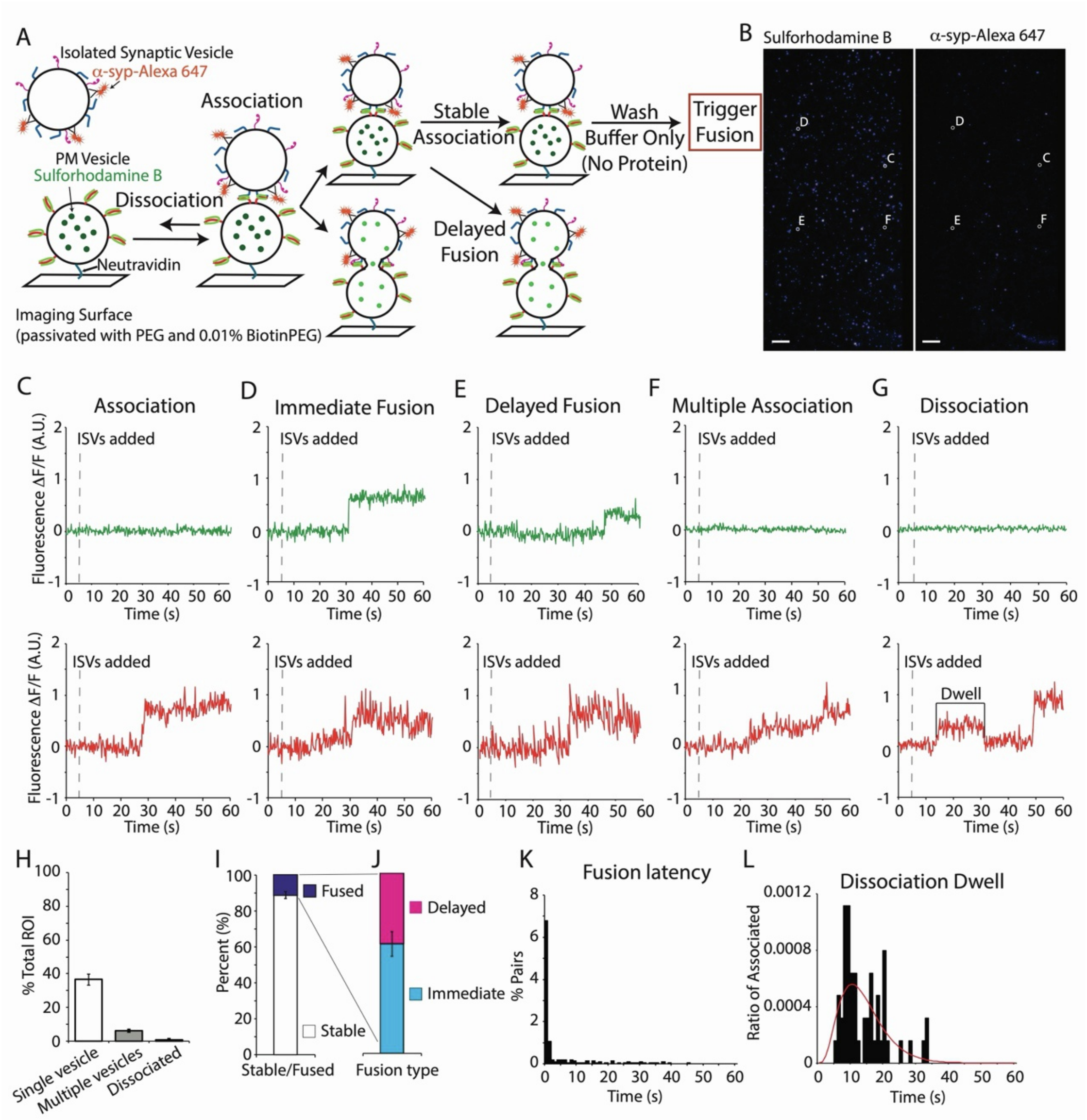

Figure S2. ISV association with PM vesicles, related to Figure 2. (**A**) Experimental scheme of the simple ISV-PM system. Plasma membrane mimic vesicles (PM Vesicles, with reconstituted syntaxin-1 and SNAP-25) were attached to the imaging surface via PEG-biotin-neutravidin-biotin-PE interactions. Labeled ISVs were then added to the imaging chamber. During association, vesicles may or may not fuse as the imaging continues. Prior to triggering fusion, the sample was washed with vesicle buffer without any protein components. (**B**) Representative fields of view. Left channel is the sulforhodamine B (fusion indicator) detection ("green") channel, right is the Alexa-647 (vesicle association) detection ("red") channel. Scale bar indicates 10 μm. Traces from example ROIs marked (**C-F**) are shown below. (**C**) Stable vesicle association. A stable stepwise increase in the red channel indicates ISV arrival, and no change in the green channel indicates no fusion has occurred. (**D**) A stepwise increase in the red channel concurrent with a stable stepwise increase in the green channel indicates that an ISV has arrived and, within 1s, fused with the PM vesicle. (**E**) Similarly, a stepwise increase in the red channel followed after some

delay by a stepwise increase in the green channel indicates delayed fusion. (**F**) Multiple ISVs could be seen associating with some PM vesicles. These associations were ignored during subsequent fusion analysis. (**G**) Rarely ISVs would associate with PM vesicles for a period followed by dissociation, a stepwise decrease in fluorescence. Note the corresponding PM vesicles were still available for a second round of association. (**H**) Quantification of association modalities. (**I**) Distribution of stable associations vs fusion events. (**J**) Distribution of fusion events as either occurring immediately or after a delay. (**K**) The fusion latency, or time between ISV arrival and fusion. (**L**) Distribution of the delay between vesicle association and dissociation. Vesicle association, dissociation, and dissociation dwell times are normalized to the total number of available PM vesicles (total ROIs). Fusion latency is normalized to the total number of vesicle pairs, determined by overlapping ROIs from the green and red channels (see methods).

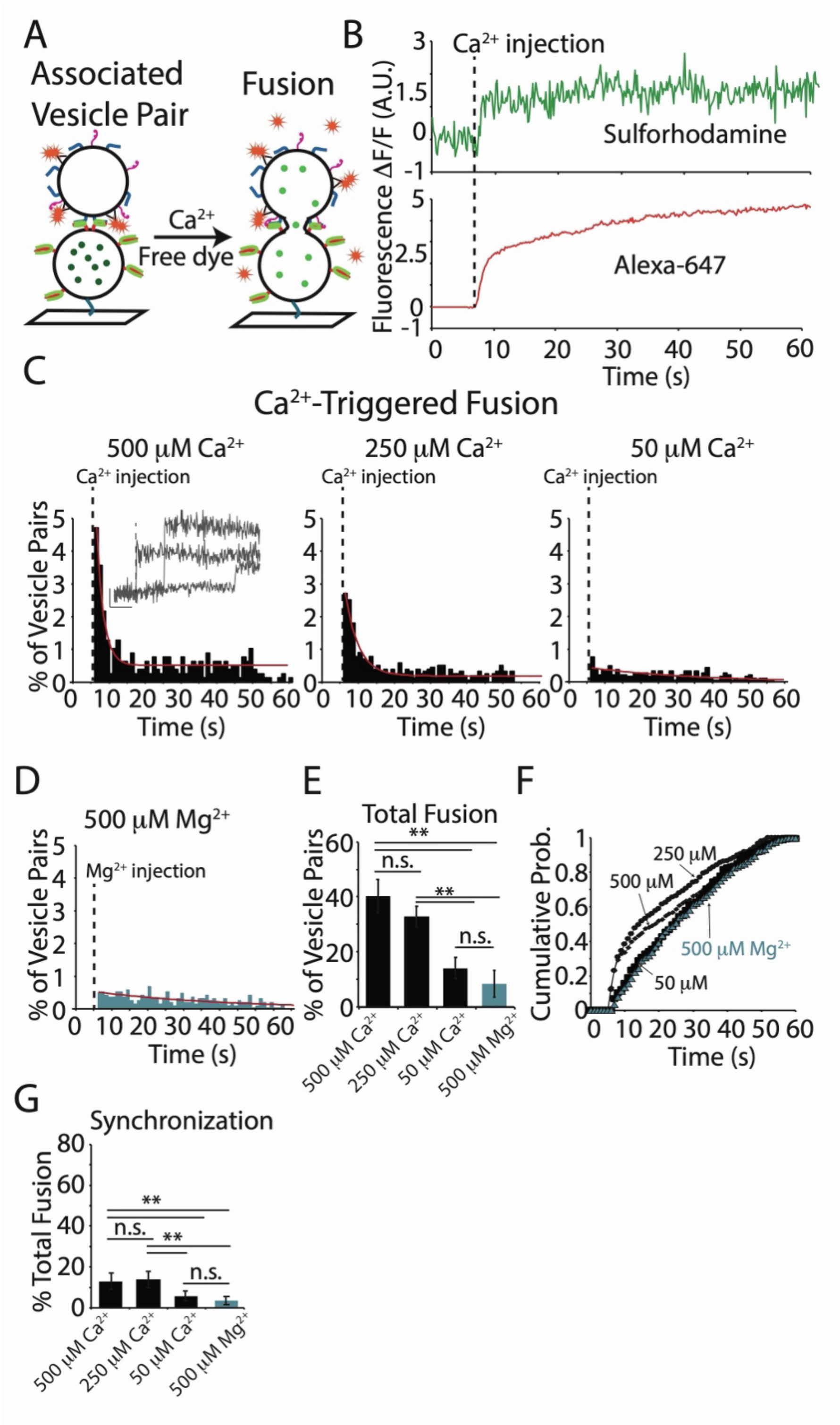

Figure S3. ISV Fusion with PM vesicles, related to Figure 2. (**A**) For the simple ISV-PM system, starting from stable ISV-PM vesicle associations, Ca^{2+} solutions together with free Alexa-647 dye were injected, and vesicle fusion was monitored. (**B**) Example normalized trace at a single ROI showing fusion as a stepwise increase in the green sulforhodamine channel and Ca^{2+} arrival as indicated by fluorescence by the free Alexa-647 dye monitored

in the red channel. (**C**) Histograms of the time of stepwise fluorescence increase in the green channel (indicating fusion) in response to a Ca^{2+} injection (marked by a vertical dashed line) at 500 μM (left), 250 μM (middle), and 50 μM (right) Ca^{2+} concentrations. The inset in the left subpanel shows example fluorescence traces of the sulforhodamine channel (scale represents a y-axis of 1.5 ΔF/F (A.U.) and an x-axis of 10 seconds). Histograms were fit by a line that was the sum of two exponential decays (for 500 μM Ca^{2+}, 0.6 s^{-1} and amplitude 0.04 and 0.02 s^{-1} with amplitude 0.007; for 250 μM Ca^{2+} 0.4 s^{-1} with amplitude 0.02 and 0.03 s^{-1} with amplitude 0.005; for 50 μM Ca^{2+} a single decay with time constant 0.03 s^{-1} and amplitude 0.004). Least sum of squares minimization was used for fits. (**D**) Injection of 500 μM Mg^{2+} elicited only minimal fusion, which was highly desynchronized (fit with single exponential decay of 0.03 s^{-1}). (**E**) Quantification of the total amount of fusion over the observation period of 60 seconds. The total fusion at both 500 and 250 μM Ca^{2+} was significantly different from 50 μM Ca^{2+} and 500 μM Mg^{2+} (analyzed with one-way ANOVA with post-hoc Tukey-Kramer test, ** means p-value < 0.005, "n.s." means no significance was observed p-value > 0.05). For panels **C-E**, vesicle fusion is normalized to the total number of vesicle pairs. (**F**) Cumulative probability of fusion shows the difference in fusion synchronicity relative to Ca^{2+} or Mg^{2+} injection (KS-test 500 μM:250 μM Ca^{2+} p-value=0.08; 50 μM Ca^{2+}:500 μM Mg^{2+} p-value=0.461; all other comparisons p-value<0.005). (**G**) The ratio of fusion events in the first second (5 acquisition frames) after Ca^{2+} arrival to all fusion events we term "synchronization". All error bars indicate the standard error for association, fusion during association and dissociation measurements for several replicates (Supplementary Table 1). For all bar graphs, a one-way ANOVA with post-hoc Tukey-Kramer test was used; one asterisk indicates a p-value <0.05, and two asterisks indicate p-value <0.005. For all cumulative probability histograms, a KS-test was performed for all pairwise comparisons.

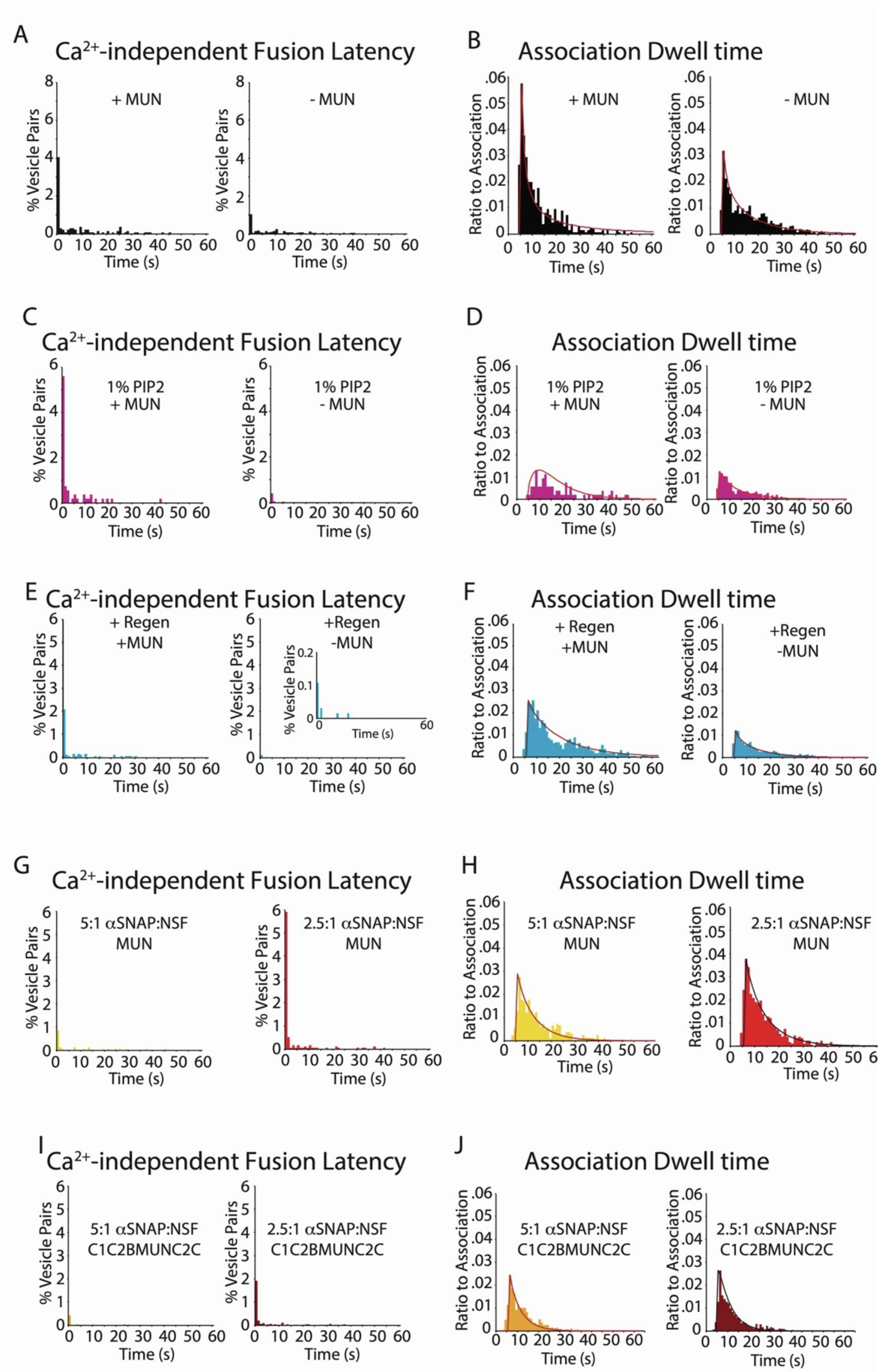

Figure S4. ISV Ca^{2+}-independent fusion and ISV dissociation latencies, related to all Figures. Distribution of Ca^{2+}-independent fusion latencies (the time between ISV arrival and fusion), left and association dwell time (duration between ISV arrival and dissociation)

right. (**A** and **B**) Conditions as in Figure 2; SM vesicles containing 3% PIP2. (**C** and **D**) Conditions as in Figure 3; SM vesicles containing 1% PIP2. (**E** and **F**) Conditions as in Figure 4; SM vesicles containing 1% PIP2 with the ATP regeneration system present. (**G** and **H)** Conditions as in Figure 5; SM vesicles containing 1% PIP2, with the ATP regeneration system comparing high 5:1 (yellow) and low 2.5:1 (red) α–SNAP:NSF ratios. (**I** and **J**) Conditions as in Figure 6; SM vesicles containing 1% PIP2, with the ATP regeneration system present using high (orange) and low (dark red) ratios of α–SNAP:NSF using the larger C1C2BMUNC2C fragment of Munc13.

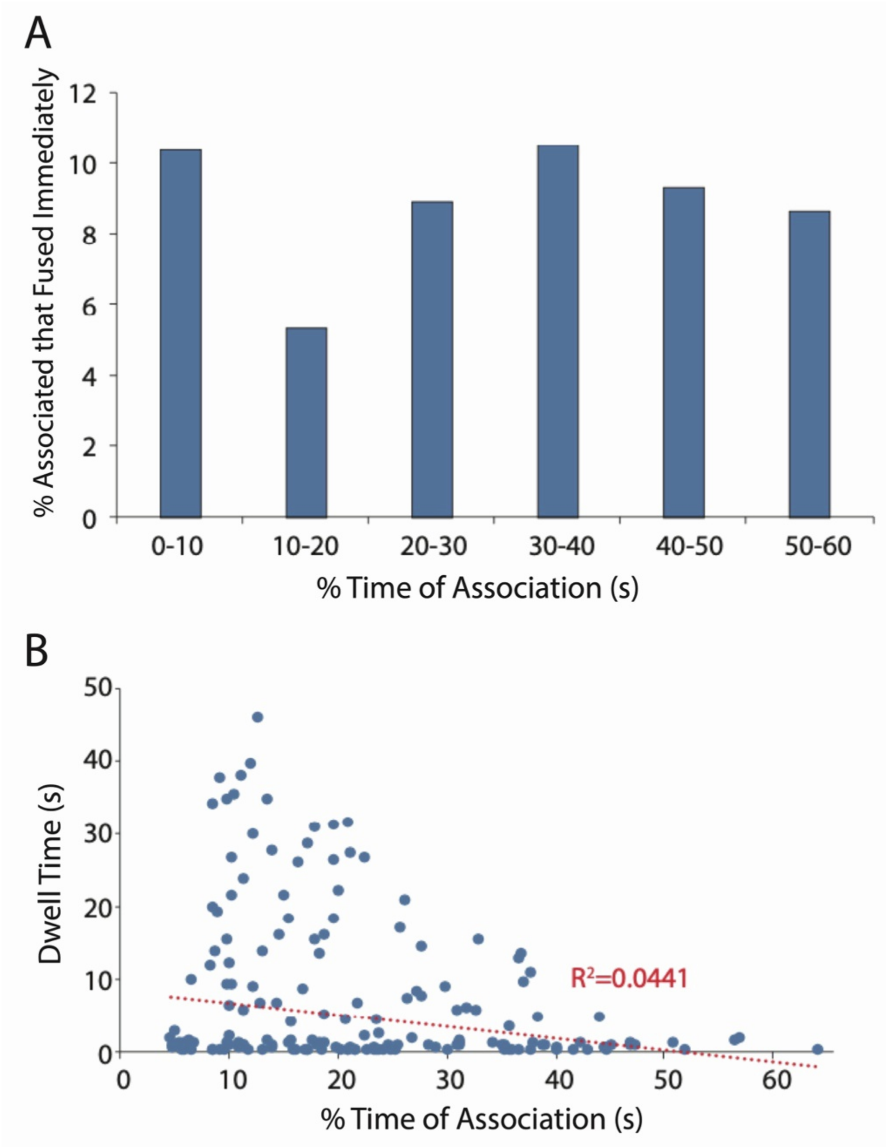

Figure S5. Ca^{2+}-independent fusion and ISV dissociation are independent of ISV association time, related to Figure 2. (**A**) Shown are the number of association events that resulted in immediate fusion normalized to the total number that docked in the same time frame. Immediate association was independent of ISV arrival time (top). (**B**) Shown are the ISV association dwell time as a function of association time. The dwell time was largely independent of ISV association time. However, image acquisition stopped shortly after 60 seconds. Therefore, long dwell time events can only be observed for early association events.

Table S1, Related to all figures and STAR Methods. Number of movies collected, experimental preparations used, total ROI analyzed, docking, Ca^{2+}-independent fusion, and undocking events observed for the specified experiments.

Experiment	Movies	Experimental preparations (# of separate LP2 preparations)	Total ROIs	Association	Fusion during association	Dissociation
Figure2B-G<br>3% PIP2	19 (MUN)<br>24 (no MUN)	6<br>6	24,673<br>31,747	1,357<br>3,863	116<br>349	472<br>1,043
Figure 3B-G<br>1% PIP2	12 (MUN)<br>16 (no MUN)	3<br>3	17,759<br>19,470	536<br>3,558	97<br>354	67<br>413
Figure 4B-G<br>1% PIP2<br>regeneration system	18 (MUN)<br>19 (no MUN)	5<br>3	53,977<br>43,491	2,233<br>6,527	24<br>19	703<br>757
Figure 5B-G<br>5:1 vs 2.5:1 α-SNAP:NSF	11 (5:1 α-SNAP:NSF)<br>24 (2.5:1 α-SNAP:NSF)	4<br>4	33,967<br>80,329	1,637<br>1,727	28<br>138	352<br>590
Figure 6B-G<br>5:1 vs 2.5:1 α-SNAP:NSF<br>C1C2BMUNC2C	9 (5:1 α-SNAP:NSF)<br>21 (2.5:1 α-SNAP:NSF)	3<br>4	29,964<br>67,256	4,958<br>4,850	36<br>149	1,013<br>851

Table S2, Related to all figures and STAR Methods. Number of movies collected, experimental preparations used, total ROI analyzed, total pairs analyzed, and total fusion counts for the specified Ca^{2+}-triggered fusion experiments.

Experiment	Movies	Experimental preparations (# of separate LP2 preparations)	Total ROIs	Total Pairs	Fusion
Figure2G-L<br>3% PIP2	8 (MUN)<br>15 (no MUN)	5<br>4	1,758<br>9,168	819<br>1,916	169<br>203
Figure 3G-K<br>1% PIP2	6 (MUN)<br>15 (no MUN)	3<br>3	905<br>5,366	377<br>1,455	217<br>179
Figure 4G-M<br>1% PIP2<br>Regeneration system	20 (MUN)<br>18 (no MUN)<br>11 (Mg2+)	6<br>3<br>3	17,946<br>17,458<br>13,031	5,365<br>5,051<br>3,054	2,021<br>338<br>226
Figure 5G-L<br>5:1 vs 2.5:1 α-SNAP	20 (5:1 α-SNAP:NSF)<br>Washout*<br>20 (5:1 α-SNAP:NSF)<br>24 (2.5:1 α-SNAP:NSF)	6<br>3<br>4	17,946<br>11,009<br>14,364	5,365<br>7,680<br>10,676	2,021<br>419<br>1,418
Figure 6G-L<br>5:1 vs 2.5:1 α-SNAP:NSF<br>C1C2BMUNC2C	9 (5:1 α-SNAP:NSF)<br>21 (2.5:1 α-SNAP:NSF)	3<br>4	22,891<br>30,355	11,330<br>16,621	1,782<br>6,505

* Reproduced from Figure3h-m
